# A Peer-Led, Narrative-Based, and Mobile-Supported Intervention in Opioid Use Disorder: Multiphase Qualitative and Longitudinal Observational Study

**DOI:** 10.2196/82485

**Published:** 2026-02-05

**Authors:** Maria C Latimer, Lydia Gregg, Mustapha Saheed, Karin E Tobin, Sharon M Kelly, Tracy Agee, R Joshua Steele, Nicholas Frankiewicz, Tarfa Verinumbe, Carl Latkin, Oluwaseun Falade-Nwulia

**Affiliations:** 1 Department of Medicine, School of Medicine, Johns Hopkins University, 5200 Eastern Ave, MFL Center Tower, Suite 381, Baltimore, MD, 21224, United States, 1 410-793-7293; 2 Department of Art as Applied to Medicine, School of Medicine, Johns Hopkins University, Baltimore, MD, United States; 3 Department of Radiology and Radiological Science, School of Medicine, Johns Hopkins University, Baltimore, MD, United States; 4 Department of Health, Behavior and Society, Johns Hopkins Bloomberg School of Public Health, Baltimore, MD, United States; 5 Applied Physics Laboratory, Johns Hopkins University, Laurel, MD, United States

**Keywords:** opioid epidemic, opioid use disorder, substance use, mobile health intervention, mHealth, mobile app, graphic narrative, graphic novel, comics, animation, mobile health

## Abstract

**Background:**

The ongoing opioid epidemic has been associated with increases in emergency department visits and hospitalizations for drug overdose and injection-related infections. These encounters with the health care system provide an opportunity to offer drug treatment linkage and support for people with opioid use disorder (OUD). There is a need for interventions that enhance linkage to and engagement in treatment with medication for opioid use disorder (MOUD) for people with OUD identified in hospital settings as they transition back to community settings.

**Objective:**

The mTools4life (Johns Hopkins University) study aimed to develop and evaluate a peer-led intervention integrating narrative-based health communication into a mobile health (mHealth) app to increase posthospitalization engagement in MOUD and reduce substance use.

**Methods:**

The formative phase of the study consisted of semistructured interviews with people with OUD and clinicians who provide care to people with OUD. Interviews sought to identify salient content to include in visual narratives within the mHealth app and information that may increase motivations for behavior change related to MOUD engagement. The intervention was developed in accordance with the information-motivation-behavioral skills model, transportation theory, and the transtheoretical model. The pilot phase of mTools4Life (Johns Hopkins University) aimed to evaluate the acceptability and usability of the intervention. People with OUD were recruited from the Johns Hopkins Hospital Emergency Department and consented to receive the intervention for a 3-month period. Participants completed a study survey at baseline and a 3-month follow-up. Data on demographics, past 30-day substance use, MOUD, and intervention appropriateness and acceptability were obtained at both time points. Additional data on intervention uptake and frequency of use were collected at follow-up. Dependent samples 2-tailed *t* tests were conducted on continuous data, and Fisher exact tests were conducted on count data.

**Results:**

Twenty people with OUD piloted the intervention. The sample was mostly male (13/20, 65%) and non-Hispanic White (13/20, 65%) with a mean age of 41.1 (SD 8.7) years. Most participants (16/20, 80%) completed the 3-month follow-up. Fewer participants reported opioid use at follow-up (9/16, 56.3%) compared to baseline (20/20, 100%; *P*=.001, and mean days of opioid use out of the past 30 days declined from baseline (19.9, SD 11.7) to follow-up (8.3, SD 11.4; *P*=.002). MOUD treatment in the prior 3 months was reported by 65% (13/20) of participants at baseline and 81.3% (13/16) at follow-up (*P*=.46). Most participants used the app (11/16, 68.8%) or engaged with their peer navigator (10/16, 62.5%) during the intervention period. At follow-up, mean acceptability and appropriateness scores (scale 0‐5; higher score indicating greater acceptability or appropriateness) were 4.5 (SD 0.5) and 4.3 (SD 0.8), respectively.

**Conclusions:**

This study demonstrates the feasibility of the development and deployment of a narrative-based mHealth intervention to support OUD care engagement and preliminary data in support of the intervention’s acceptability, appropriateness, and effectiveness

## Introduction

Over the past 2 decades, the opioid epidemic has been accompanied by dramatic increases in drug overdose deaths, peaking at over 100,000 overdose deaths in 2023 [1]. Similarly, injection-related infections such as HIV, hepatitis B virus, hepatitis C virus, and life-threatening bacterial and fungal infections have increased, leading to skin and soft tissue infections, osteomyelitis, endocarditis, and sepsis [2-8]. The rise in infections related to opioid use is associated with increases in hospitalizations, as demonstrated by an estimated increase in admissions for injection-related endocarditis in the United States from 2900 admissions in 2013 to greater than 20,000 admissions in 2017 [2] and increases of 12%‐35% for injection-related infections from 2016 to 2018 in Michigan [8]. Nationally, infective endocarditis-related mortality increased an average of 5% (95% CI 3-8) per year for people aged 25- 34 years and 2% (95% CI 1-3) per year for people aged 35-44 years from 1999 to 2020 and was associated with an increase in substance use disorder (SUD) as an underlying cause of death in both age groups [9]. These infections, in addition to nonfatal overdose injuries, have resulted in people who use drugs interfacing more frequently with the hospital system [7,10].

Prior studies show that people who use drugs are less likely to engage in outpatient health care, with stigma and previous negative encounters with health care providers and systems, cost, unstable housing, and unreliable transportation frequently cited as barriers to seeking care [11-14]. Fear of withdrawal symptoms and treatment side effects may also reduce willingness to seek medication treatment [11,15]. Consequently, people who use drugs are more likely to seek emergency care than the general population [16-18]. Emergency department (ED) encounters with or without subsequent hospitalization provide an opportunity to offer opioid use disorder (OUD) care and encourage ongoing engagement in OUD care after ED or hospital discharge [19]. 

Treatment of OUD with buprenorphine or methadone reduces opioid use, improves quality of life, and reduces opioid-related morbidity and mortality [20-24]. Several studies have demonstrated high patient acceptability and effectiveness of medication for opioid use disorder (MOUD) prescription in the ED [25-28]. MOUD uptake and retention in community settings, however, remain low [29-31]. Interventions linking people to MOUD that also support retention in community-based treatment are needed. The use of peer recovery support services (PRSS) is an approach to retention that provides support to challenge stigmatizing beliefs about long-term use of MOUD and offers relatable modeling for self-efficacy and behavior change. Peer navigators support patients by helping them interface with the medical system in productive and affirming ways while sharing their own stories of recovery. Peer navigation by individuals with lived experience has been associated with success in linking people with OUD in acute care settings to community MOUD, reducing substance use, increasing retention in OUD care, and reducing ED and inpatient hospital readmission [32-34].

Since peer navigators leverage their own narratives of recovery while supporting clients, they are a natural extension of and counterpart to mobile health (mHealth)–delivered narrative health communication. Integration of narratives for health communication into mHealth tools may further expand the reach of narratives as a health information tool and provide additional resources for the intended behavior change of SUD care engagement [35-40]. Therefore, the mTools4life study sought to develop and evaluate a peer-based intervention integrating narrative-based health communication into a mHealth app to increase posthospitalization engagement in MOUD through (1) formative work with people with OUD and service providers and (2) development, pilot, and evaluation of the intervention. Specifically, the pilot phase sought to examine the acceptability and appropriateness of the intervention and reveal any changes in patterns of MOUD engagement or substance use.

## Methods

### Study Design and Setting

The mTools4Life study was a multiphase formative and exploratory research study that aimed to develop and evaluate an intervention to enhance linkage to care for people with OUD through a peer support-based and narrative-centered intervention with mobile phone support. The core intervention consists of a peer navigator trained in PRSS and a guided introduction to the mTools4Life mobile app.

The formative phase of the study included semistructured interviews with 4 people with OUD and 4 care providers to people with OUD, which aimed to identify (1) key moments in substance use and recovery to include in the narratives, (2) salient information that may increase motivations for long-term use of MOUD, and (3) perspectives on barriers and facilitators of OUD treatment engagement to address in the mobile app. Interviews were thematically analyzed by reviewing transcripts for recurring ideas and grouping the ideas into broad themes that reflected shared experiences and perspectives. The qualitative interview results were used to develop the intervention, including the creation of narratives and content embedded in the mobile app. After deployment, mTools4Life underwent pilot usability and acceptability testing. People with OUD were recruited from the Johns Hopkins Hospital (JHH) ED and consented to receive the intervention for a 3-month period. Participants provided informed consent and completed a study survey at baseline and at a 3-month follow-up.

### mTools4Life App

Based on the identification of food insecurity, homelessness, transportation, and employment challenges as barriers to substance use recovery during the formative phase, tools aimed at addressing these issues were embedded in the mobile app. The app is downloadable for Apple and Android smartphones and includes five main sections: (1) My Health, which consists of peer navigator contact information, medication reminders, appointment reminders, and a withdrawal symptom tracker; (2) Resources; (3) Chat, to easily connect with a peer navigator by text or phone call; (4) Stories, which depicts two individuals’ recovery journeys; and (5) the Crisis Hotline. The Resources section includes access to a list of geolocated community resources in Baltimore City, broken down into 5 categories, such as food assistance, housing assistance, employment assistance, transportation assistance, and syringe exchange. Syringe exchange was included as a resource for substance use harm reduction. Short motivational messages developed in collaboration with peer navigators and clinical providers of substance use care intended to promote self-efficacy and motivation for seeking treatment for OUD were embedded in the app and automatically deployed every other day.

### Design of Narratives

The mTools4Life intervention development is guided by the information-motivation-behavioral skills model (IMB) for health behavior change and transportation theory. The IMB model asserts that provision of information can drive motivation and increase self-efficacy in behavioral skills, and the interactions of these constructs can evoke behavior change [41]. Various interventions, including mHealth apps, based on the IMB model have produced lasting behavior change [42,43]. The IMB model is often used as a foundation for mHealth interventions [44]. Providing information in narrative format has been shown to be an effective approach to enhancing health communication to promote behavior change [45]. Health communication through storytelling of health seeking, care access, and the recovery journey from individuals with a lived experience of substance use and recovery (ie, peer navigators) may be particularly effective as a communication tool for sustained health behavior change [46,47]. Transportation theory describes the sensation of being mentally immersed in the imagery of a narrative. Transportation has the potential to significantly impact behavior change through the reduction of cognitive counterarguments, new connections with characters who are successful at obtaining OUD care, vivid mental imagery, and emotional engagement with the story [48-50].

**Figure 1.**
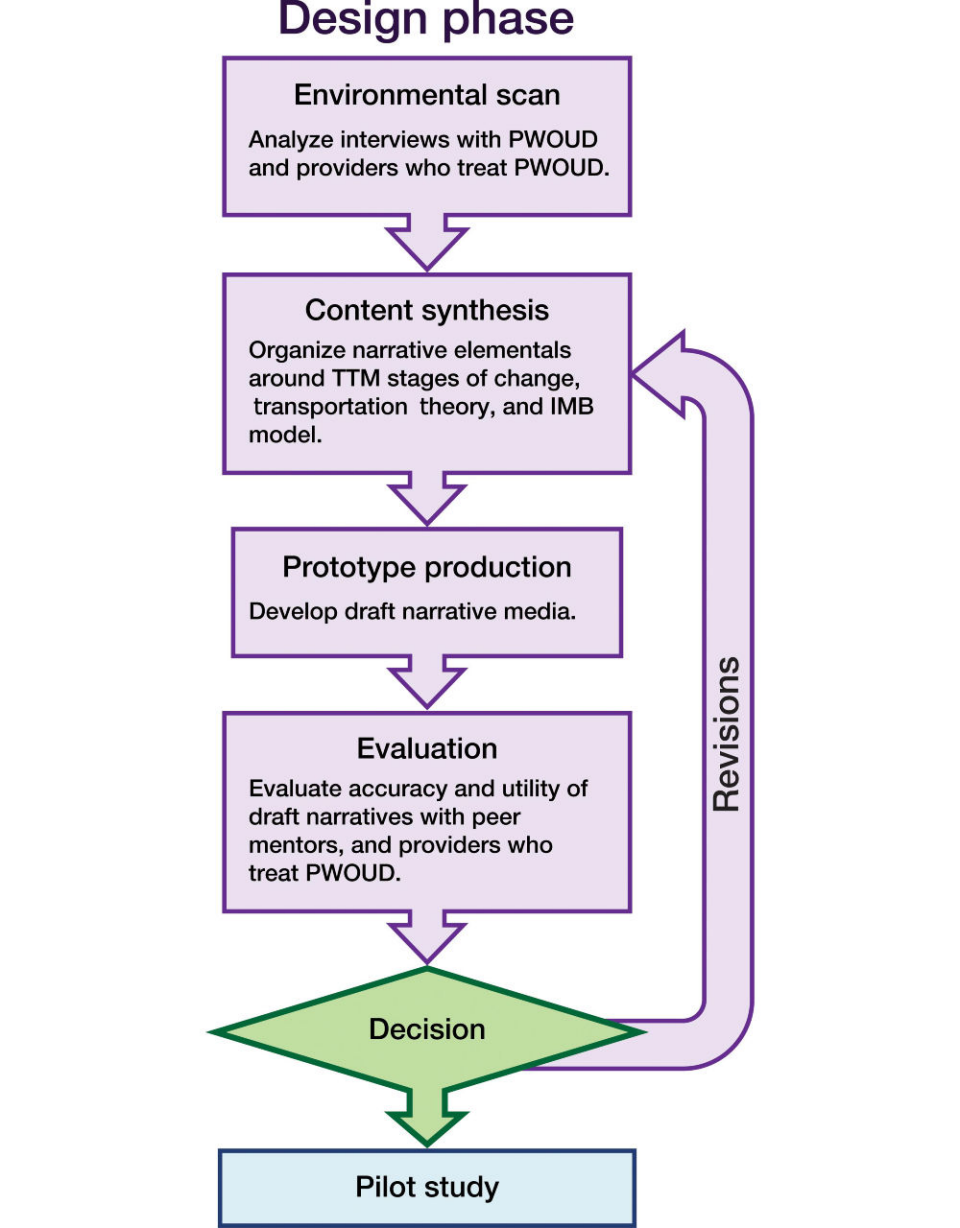
The modified version of Roozenburg empirical design cycle that was used in the process of developing the mTools4Life narratives. IMB: information-motivation-behavioral skills model; PWOUD: people with opioid use disorder; TTM: transtheoretical model.

Roozenburg empirical design cycle [51,52] was used to guide the iterative process of developing the mTools4Life narratives (Figure 1). The narratives were created based on the data collected from semistructured interviews conducted in the Formative Phase and discussions with 2 peer navigators and a nurse practitioner involved in treating patients with OUD. The transtheoretical model (TTM) provided a foundation for organizing narrative elements around stages of change [53] (Multimedia Appendix 1). Decisional balance, a construct of TTM, represents an individual’s assessment of the pros and cons of change as they transition through the precontemplation, contemplation, action, and maintenance stages [53]. Interviews were analyzed qualitatively for factors that were identified by participants as crucial to the decision to seek treatment for OUD or remain in recovery, including attitudes and beliefs, barriers to change, and information needed to pursue treatment. These factors, along with additional events and experiences documented in the interviews, were organized into pros and cons (Multimedia Appendix 2) and then reframed as exemplar events and experiences (Multimedia Appendix 1). The exemplars were grouped according to the TTM stage to which they best corresponded. For example, an exemplar experience of learning that taking buprenorphine is not “just trading one drug for another” could promote a character’s transition from a state of ambivalence to having a desire for change as they contemplate the pros and cons of treatment. This exemplar was, therefore, placed under the contemplation stage in TTM.

**Figure 2.**
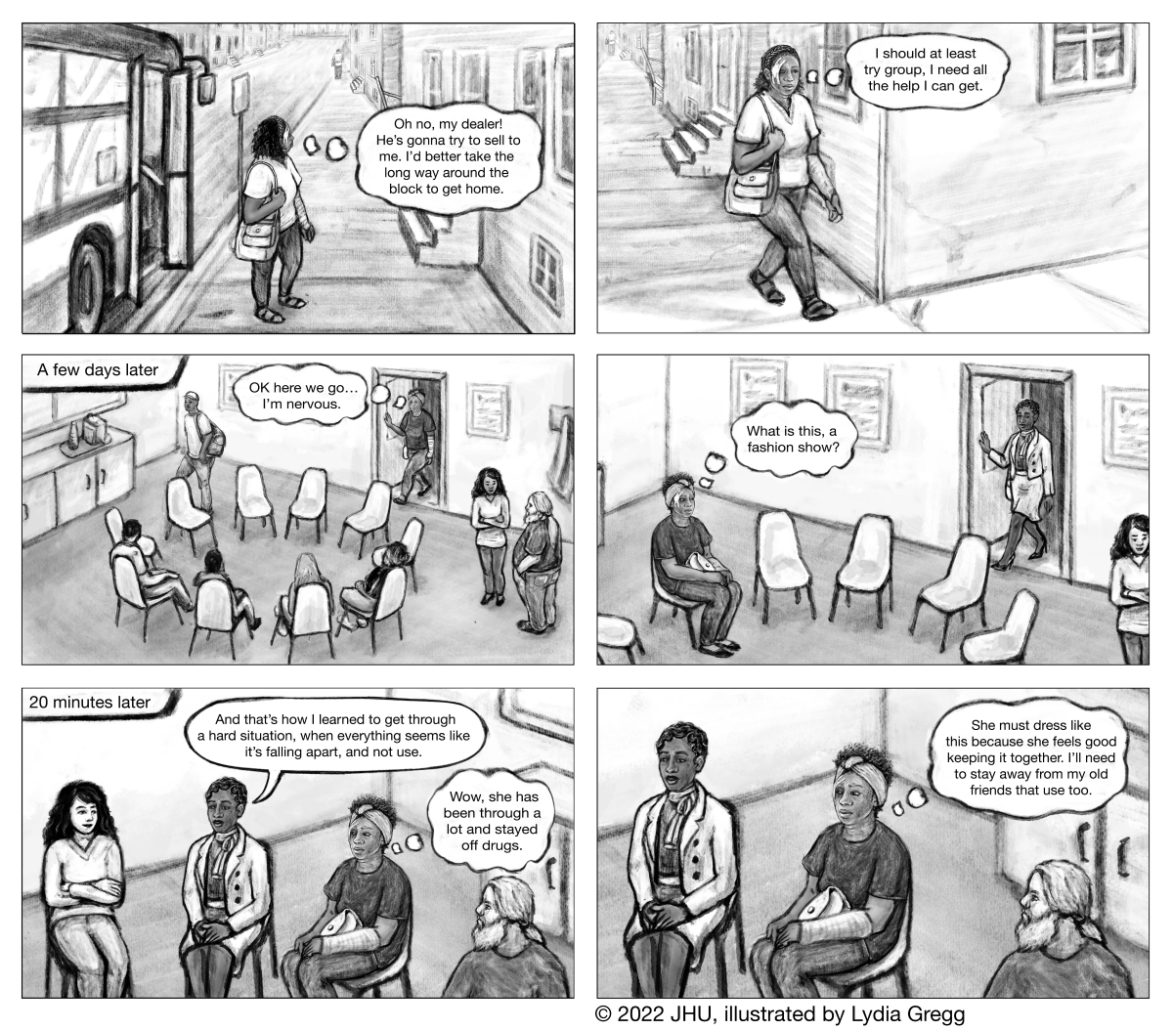
Representative images from Eva’s Story, one of two visual narratives included in the story.

The exemplars were then combined and arranged into 4 narratives that represented different races and genders. Each narrative followed the structure of TTM, which creates an opportunity for a narrative arc as the main character takes action and faces challenges during each stage, experiencing rises and declines in positive emotions typical of popular narratives [54]. In accordance with the IMB model, the stories provided health information relevant to OUD treatment and were intended to promote self-efficacy related to a personal recovery journey, thereby increasing motivation for behavior change. The narrative characters model self-efficacy as they move through stages of TTM. For example, one character believed that modifying her normal path home could help her avoid her drug dealer and avoid drug use (Figure 2).

In accordance with transportation theory, the narratives were designed with consideration for features of stories that can positively affect transportation levels, including identifiable, relatable characters; an imaginable, vivid plot; and verisimilitude of the narrative events [55]. For the recipient, the level of attention paid, familiarity with the subject, their demographics, and situational factors surrounding the expectations of the narrative can affect the level of transportation into a story [55]. To address these recipient-related factors, peer navigators helped familiarize participants with the app’s purpose and features during study enrollment.

Of the 4 written narratives, 2 were chosen for full development into the graphic narrative format (ie, graphic novels, and comics; Figures2  3, MultimediaAppendices 3  4). The narratives were illustrated using Adobe Photoshop (Adobe Systems) by a medical illustrator with graduate-level training and experience working in this format. Simple animations (MultimediaAppendices 5  6) consisting of fades between images and character voiceovers were created with Adobe After Effects. The graphic narratives and animations were incorporated into the “Stories” section of the app.

**Figure 3.**
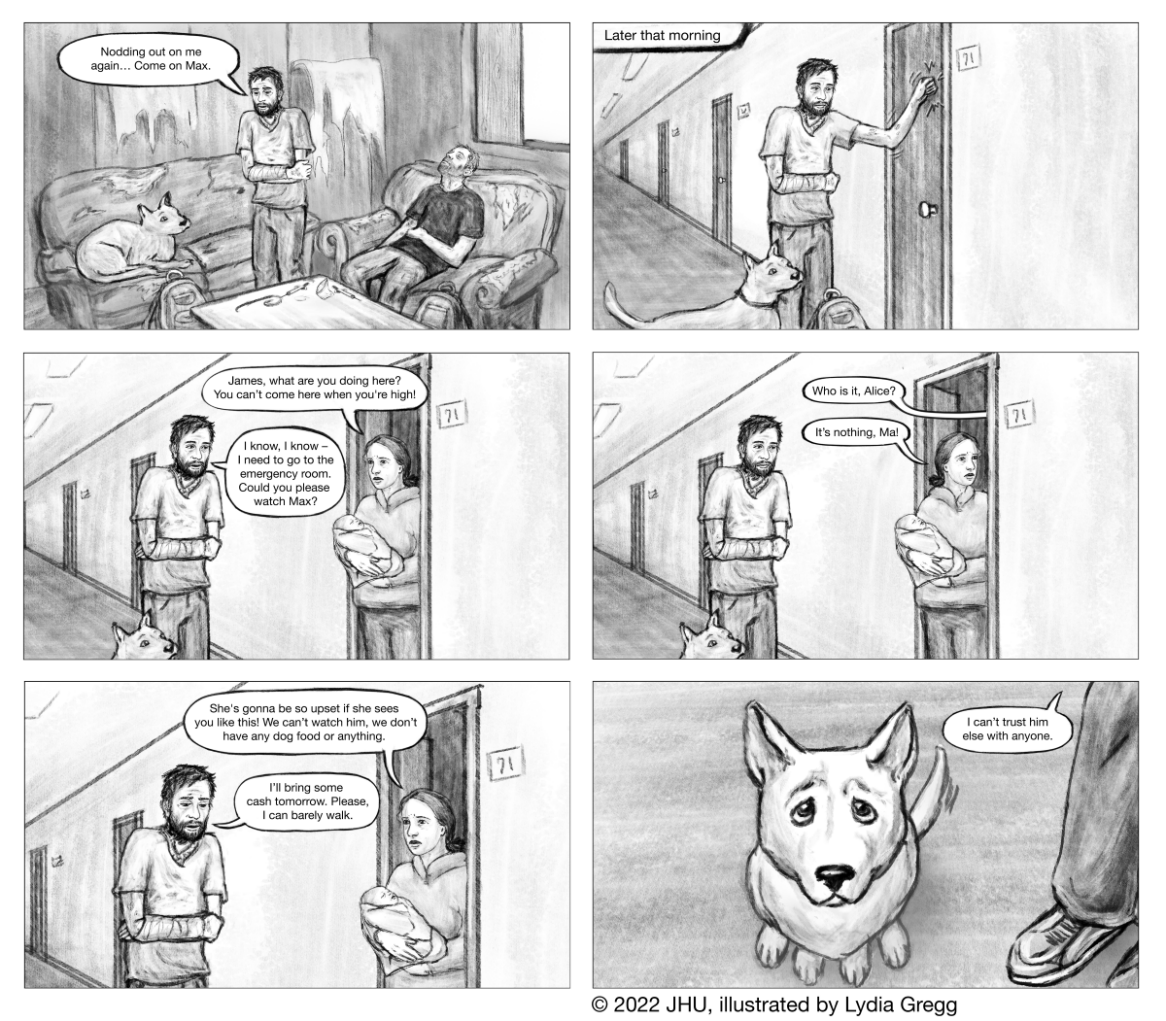
Representative images from James’s Story, one of two visual narratives included in the story.

### Peer Navigators

Three peer navigators with previous experience with OUD peer recovery support within outpatient and inpatient care settings provided support to participants, including through sharing their own personal narratives of recovery. Peer navigators provided support based on the principles of Recovery Support Services, including harm reduction, nonjudgment, person-centered support, and self-advocacy [56]. Peer navigators were trained to support study participants in downloading the mTools4Life app and demonstrated use of the app. Peer navigators used a participant engagement protocol with an expectation for at least weekly outreach either by phone or in person. Contacts were recorded in a spreadsheet and were considered successful if contact was achieved (ie, contact was reciprocal) or unsuccessful if contact attempts were not returned (eg, text was unanswered, phone call was unanswered, and voicemail did not receive a response).

### Pilot Phase Procedures

Patients accessing care at the JHH ED between April and August 2022 were recruited for participation in the pilot phase. Inclusion criteria were (1) presentation to the ED with a substance use-related condition (eg, cellulitis, overdose, or wound care), (2) any use of illegal opioids or opioids not as prescribed in the previous 30 days, (3) English-speaking, and (4) possession of a smartphone capable of downloading the mTools4Life app. Patients were excluded if they were unable or unwilling to engage in study procedures.

Patients were referred for study participation either by ED staff (eg, clinicians, nurses, and social workers) or through assessment of potential eligibility based on chart review conducted by research staff. A research coordinator met with eligible patients, obtained informed consent, administered baseline assessments, and connected enrolled participants to an assigned peer navigator. Baseline assessments consisted of questions about demographics, employment, substance use, readiness for and confidence in their ability to reduce or cease substance use, physical and mental health, stigma, social support, and acceptability and appropriateness of the mTools4Life intervention.

Upon completion of the baseline assessments, participants were assigned to one of three peer navigators who met with participants either prior to ED discharge or in the community after discharge. Peer navigators were available by phone or in person from 8:30 AM to 5:00 PM, Monday through Friday. The number and purpose of engagements with participants were recorded.

Participants were contacted by research staff 3 months post enrollment and scheduled to complete a follow-up assessment. The follow-up assessment included the baseline questions about substance use and MOUD, as well as questions asking about participants’ uptake of and satisfaction with the intervention, described more in the following section.

### Pilot Phase Measures and Analyses

The primary outcomes of interest were engagement in MOUD treatment in the past 3 months (yes or no) and past 30-day substance use (yes or no). Secondary outcomes included the number of days of substance use in the past 30 days, the importance of, confidence in, and readiness for cutting down or quitting substance use, and the acceptability of, appropriateness of, and satisfaction with the intervention. All outcomes were assessed by self-report on a questionnaire.

Participants were asked 3 questions at each time point to assess the importance of, confidence in, and readiness for making a change (cutting down or quitting) in their substance use. Responses were measured on a scale of 1-10, where 1 means not at all (important, confident, and ready) and 10 means extremely (important, confident, and ready). These tools have generally been found to predict behavior change in studies of people who use drugs and other substances [57-59].

Two four-item scales, the Acceptability of Intervention Measure (AIM) and the Intervention Appropriateness Measure (IAM), were used to evaluate acceptability and appropriateness of the intervention [60]. Participants rated a series of statements about the intervention’s acceptability and appropriateness for connecting people to treatment for OUD from 1 (“Completely disagree”) to 5 (“Completely agree”). We calculated participants’ average score for each measure out of 5. A participant was determined to have rated the intervention as acceptable or appropriate if their average score was greater than 3.

Participant evaluation of peer navigators was measured using the Texas Christian University (TCU) Treatment Engagement form questions on Counseling Rapport [61]. This 11-item tool was modified to replace the word “counselor” with “peer navigator.” The total score for this scale ranges from 11 to 55, with higher scores indicating greater rapport.

Data on mobile app usage were self-reported by participants. Satisfaction with the app was measured using questions from the mHealth App Usability Questionnaire (MAUQ) [62], the Usefulness, Satisfaction, and Ease of Use (USE) questionnaire [63], and the Telehealth Usability Questionnaire (TUQ) [64]. Participants were asked 5 questions on usability, 9 on usefulness, and 8 on satisfaction and rated their agreement with each statement on a scale of 1 (“Disagree”) to 7 (“Agree”). Total scores range from 5 to 35 for the usability subscale, from 9 to 63 for the usefulness subscale, and from 8 to 56 for the satisfaction subscale. Four statements rated on the 1 (“Disagree”) to 7 (“Agree”) scale were used to measure transportation into the narratives, 3 of which came from the Transportation Scale–Short Form (TS–SF) [65]. Relatability to the characters was measured with the statement, “I could relate to the characters in the stories in the app,” modified from the original wording, “While reading the narrative I had a vivid image of [character name].” [65] Higher scores indicated greater usability, usefulness, and satisfaction with the app and transportation into the narratives.

Frequencies and means were obtained to describe sociodemographic characteristics and the outcomes. Fisher exact tests were conducted to assess the differences in the number of participants who reported using substances and who reported receiving MOUD at follow-up compared to baseline. Dependent samples 2-tailed *t* tests were conducted on paired data to examine the difference in the number of days of substance use in the past 30 days at baseline and follow-up (n=16). All analyses were conducted using R version 4.3.1 (R Foundation for Statistical Computing).

### Ethical Considerations

The study was approved by the Johns Hopkins University School of Medicine Institutional Review Board (IRB 00220706). All participants provided informed consent. Data were deidentified. Participants received US $25 for completing the follow-up assessment.

## Results

### Formative Phase

MultimediaAppendices 1  2 summarize interview data analyzed during the formative phase, which were used to design the narratives. Both provider and patient opinions on the format of the narratives varied. Patient participants expressed that a mixture of different formats would be useful, given that individual preferences vary. Some pointed to animation as the best option, citing potentially low attention spans and low literacy levels among participants. Concerning the narrative content, the majority of patients expressed that narratives should not include any directives or patronizing content about the consequences of continued drug use. They generally expressed that there should be an emphasis on the benefits that treatment can bring to someone’s life and that withdrawal is not as painful with MOUDs. Both provider and patient participants identified smartphone delivery of the content as a potential problem, noting that many people with OUD don’t have cell phones and that many inpatient treatment facilities don’t allow patients to bring their cell phones.

### Pilot Phase: Study Sample

Overall, 219 people seen in the JHH ED for a substance use–related condition were referred for study participation during the recruitment period. Of 219, 14 declined to participate, 180 were screened and excluded for not meeting eligibility criteria, and 5 were enrolled but did not complete the baseline assessment. The most common reasons for noneligibility included not having a smartphone (n=85/180; 47.2%) and no opioid use in the past 30 days (n=62/180; 34.4%). The final sample of 20 participants was predominantly male (13/20, 65%) and Non-Hispanic White (13/20, 65%) (Table 1). The sample had a mean (SD) age of 41.1 (8.7) years. Half (n=10) of the participants reported being homeless in the preceding 3 months. Most participants (n=16/20, 80%) completed the 3-month follow-up interview.

**Table 1.** Demographics of study sample (N=20).

Characteristic	Value
Age, mean (SD)	41.1 (8.7)
Male, n (%)	13 (65)
Race and ethnicity, n (%)	
Non-Hispanic White	13 (65)
Non-Hispanic Black	5 (25)
Hispanic or Latino	1 (5)
Other	1 (5)
Phone operating system, n (%)	
Android	16 (80)
Apple	4 (20)
Homeless, past 3 months, n (%)	10 (50)
Health insurance, n (%)	
Medicaid	19 (95)
Medicare	2 (10)
Other (military or private)	2 (10)
Highest education attainment, n (%)	
Less than high school	2 (10)
High school diploma	8 (40)
Beyond high school	10 (50)
Relationship status, n (%)	
In a committed relationship	6 (30)
Separated, divorced, widowed, single	14 (70)
Infectious diseases, n (%)	
HIV infection	2 (10)
Ever diagnosed with HCV^a^	8 (40)
Opioid overdose, n (%)	
Any history of opioid overdose	12 (60)
Opioid overdose in past 90 days	3 (15)
Injection drug use in past 90 days, n (%)	14 (70)
Opioid use in the past 30 days, n (%)	20 (100)
Cocaine use in the past 30 days, n (%)	14 (70)

a HCV: hepatitis C virus.

### Substance Use and Readiness to Change

All participants reported opioid use in the past 30 days at baseline (20/20, 100%). At follow-up, opioid use declined to 56.3% (9/16; *P*=.001; Table 2). Mean days of opioid use in the past 30 days declined from 19.9 (SD 11.7) to 8.3 (SD 11.4; *P*=.002; Table 3). Cocaine was used by 70% (14/20) of participants at baseline and 37.5% (6/16) of participants at follow-up (*P*=.09). Mean days of cocaine use declined from 13.3 (SD 12.2) to 4.8 (SD 9.4) in the past 30 days (*P*=.005).

**Table 2.** Substance use in the past 30 days at baseline and 3-month follow-up (N=20).

Substance	Frequency at baseline, n (%)	Frequency at follow-up (n=16), n (%)	OR^a^ (95% CI)	*P* value
Any opioid	20 (100)	9 (56.3)	∞(2.5-∞)	.001
Heroin	17 (85)	9 (56.3)	4.2 (0.7-31.6)	.07
Cocaine	14 (70)	6 (37.5)	3.7 (0.8-19.7)	.09
Cannabis	9 (45)	6 (37.5)	1.4 (0.3-6.5)	.74
Alcohol	4 (20)	3 (18.8)	1.1 (0.2-8.7)	≥.99

a OR: odds ratio.

**Table 3.** Days of substance use during past 30 days (n=16).

Substance	Baseline, mean (SD)	Follow-up, mean (SD)	*t* value (*df*)	*P* value
Any opioid	19.9 (11.7)	8.3 (11.4)	−3.7 (15)	.002
Cocaine	13.3 (12.2)	4.8 (9.4)	−3.3 (15)	.005
Cannabis	3.3 (5.2)	2.8 (7.5)	−0.3 (15)	.77
Alcohol	2.4 (7.5)	2.2 (7.5)	−0.5 (15)	.59

The mean score for perceived importance of making a change (quitting or cutting down) in substance use increased from 9.1 (SD 1.1) at baseline to 9.8 (SD 0.5) at 3-month follow-up on a 10-point scale (*P*=.009). Participant confidence in perceived ability to make a change in substance use had a mean score of 7.6 (SD 2.5) at baseline and a mean score of 8.4 (SD 2.2) at follow-up (*P*=.13). At baseline, readiness to make a change in substance use had a mean score of 8.6 (SD 2.6) and a mean score at follow-up of 8.8 (SD 2.1; *P*=.73).

### Substance Use Treatment

At baseline, 13/20 (65%) participants had received MOUD treatment in the previous 3 months, including 55% (11/20) who received methadone, 15% (3/20) buprenorphine, and 5% (1/20) long-acting naltrexone. At follow-up, 13/16 (81.3%) participants who completed the 3-month follow-up assessment reported receipt of MOUD in the previous 3 months, with 62.5% (10/16) who received methadone and 37.5% (6/16) who received buprenorphine. Of the 13 participants who reported recent MOUD treatment at follow-up, 11/16 (84.6%) also reported MOUD use in the 3 months preceding baseline. There was no significant difference in the number of participants who received MOUD in the 3 months preceding baseline and follow-up (*P*=.46).

### Acceptability and Appropriateness of Intervention

At baseline, the mean AIM scale score was 4.1 (SD 0.4; 5-point scale), and 94.7% (18/19) rated the intervention as acceptable (score of >3; Table 4). At follow-up, the mean AIM score was 4.5 (SD 0.5), and 100% (16/16) of participants rated the intervention as acceptable.

**Table 4.** Acceptability and appropriateness of intervention ratings.

Measure	Baseline		Follow-up	
	Mean score (SD)	Participants who answered agreed or completely agreed (n=19), n (%)	Mean score (SD)	Participants who answered agreed or completely agreed (n=16), n (%)
Acceptability of Intervention Measure total score	4.1 (0.4)	—^a^	4.5 (0.5)	—
The intervention meets my approval.	4.2 (0.7)	18 (94.7)	4.3 (0.5)	16 (100)
The intervention is appealing to me.	4.0 (0.8)	17 (89.5)	4.6 (0.5)	16 (100)
I like the intervention.	4.3 (0.5)	19 (100)	4.6 (0.5)	16 (100)
I welcome the intervention.	4.1 (0.5)	18 (94.7)	4.4 (1.0)	14 (87.5)
Intervention Appropriateness Measure total score	4.0 (0.4)	—	4.3 (0.8)	—
The intervention would fit my needs.	3.9 (0.7)	16 (84.2)	4.3 (0.9)	14 (87.5)
The intervention feels suitable to me.	4.1 (0.3)	19 (100)	4.2 (0.8)	14 (87.5)
The intervention seems like a good match.	4.1 (0.3)	19 (100)	4.3 (0.8)	15 (93.8)

a Not applicable.

Regarding the appropriateness of the intervention, the mean of IAM scale score at baseline was 4.0 (SD 0.4), and 100% (19/19) of participants rated the intervention as appropriate. At follow-up, the mean of the IAM score was 4.3 (SD 0.8), and 93.8% (15/16) of participants rated the intervention as appropriate.

### Intervention Uptake

At follow-up, 11/16 (68.8%) participants reported using the app at least once since baseline. Reasons for not using the app included (1) forgetting about the app (n=3), (2) losing phone (n=2), (3) losing access to or not being able to access the app (n=1), and (4) forgetting how to use the app (n=1).

Overall, 10/16 (62.5%) participants reported engaging with the peer navigator at least once following the baseline assessment. Reasons for not engaging with the peer navigator included: (1) lost cell phone or cell service (n=2), (2) forgot about the peer navigator (n=2), and (3) not interested in peer support (n=1). More than half of participants (9/16, 56.3%) reported engagement with both the app and peer navigator, while 4/16 (25%) participants engaged with neither; 2/16 (12.5%) participants used the app but did not engage with the peer navigator, and 1 (6.3%) participant engaged with the peer navigator but did not use the app. All participants who engaged with their peer navigator rated their satisfaction with their assigned peer navigator at follow-up. The mean satisfaction score was 51.7 (SD 3.5) out of a total score of 55. Multimedia Appendix 7 describes the association of intervention engagement with selected behavioral outcomes.

Participants who reported engaging with the intervention were asked about the frequency of using each component. The most frequently used aspect of the intervention was communication with the peer navigators, with a mean score of 2.6 (SD 0.7; scale 0-5, higher scores indicating closer to daily use). App use had a mean score of 1.9 (SD 0.7).

Of the app components, the stories (mean 1.7, SD 0.8) and the resource list (mean 1.4, SD 0.9) were used most frequently, followed by medication reminders (mean 1.0, SD 1.4) and appointment reminders (mean 0.9, SD 1.2). No participants reported using the app to record symptoms of withdrawal.

Throughout the 3-month follow-up phase, peer navigators recorded a total of 234 contacts or contact attempts, of which 141 (60.3%) were successful and 93 (39.7%) were unsuccessful. Peer navigators recorded a mean of 11.7 (SD 5.0) contacts per participant. Each participant had a mean of 7.1 (SD 4.7) successful or reciprocal contacts and 4.7 (SD 3.4) unsuccessful or unanswered contact attempts from their peer navigator. Most contacts (n=125, 53.4%) were via phone call, 65 (27.8%) contacts were in-person, with the rest of the contacts (n=25, 10.7%) through text message, the mTools4Life app (n=5, 2.1%), Zoom (Zoom Communications, Inc; n=1, 0.4%), letter (n=1, 0.4%), or other (n=12, 5.1%). The primary purpose of most of the contacts was outreach (n=164, 70.1%), followed by coaching and mentoring (n=34, 14.6%). The remaining contacts had the primary purpose of appointment schedule or reminder (n=4, 1.7%), engagement with mTools4Life app (n=6, 2.6%), housing support (n=1, 0.4%), link to health care (n=2, 0.9%) or SUD treatment (n=7, 3.0%), or other (n=16, 6.8%).

### Usability and Usefulness of App

Responses on the usability and usefulness of the app were available for the 11/16 (68.7%) study participants who reported using the app at follow-up. The mean rating of the usability of the app was 33.9 (SD 1.7) out of a total score of 35. The mean usefulness rating was 59.7 (SD 5.2) out of a total score of 63, and the mean satisfaction rating was 54.8 (SD 2.1) out of a total score of 56. Participants who engaged with the stories (n=10) generally reported favorable responses related to transportation into the narratives (Table 5). The mean rating for all transportation-related statements was 5.9 (SD 1.8) out of 7 total points.

**Table 5.** Agreement with statements about stories (n=10).^a^

Statement	Value, mean (SD)
I could relate to the characters in the stories in the app.^b^^,c^	6.4 (1.1)
I was mentally involved in the stories while watching them.^d^	5.9 (2.1)
I wanted to learn how the stories ended.	5.8 (2.1)
The stories affected me emotionally.	5.5 (2.0)

a Four statements were used to measure transportation into the narratives on a scale from 1 (“Disagree”) to 7 (“Agree”).

b Relatability of the characters was measured with a modified statement.

c n=9 due to missing data.

d n=8 due to missing data.

## Discussion

### Principal Findings

In this study, we demonstrated successful development and deployment of a peer-supported, narrative-based, and mobile-supported intervention with preliminary evidence of effectiveness as demonstrated by reduction in substance use at 3-month follow-up compared to baseline. To the authors’ knowledge, this is the first study to evaluate narratives embedded into a mobile app to address SUD treatment engagement.

While a higher proportion of participants reported MOUD at 3-month follow-up compared to baseline (13/16, 81.3% vs 13/20, 65%), these differences are difficult to interpret due to the small sample size and the loss to follow-up. In addition, most of those who engaged in MOUD at follow-up also reported MOUD engagement at baseline (11/13, 84.6%), indicating that they may have already been open to MOUD treatment. The reduction in overall substance use and in paired analysis among participants with both baseline and follow-up data is reassuring and provides some preliminary evidence of intervention effectiveness.

The intervention was judged by study participants to be highly acceptable and appropriate. The component of the intervention that participants reported using most frequently was engaging with the peer navigators. In a recent study, Szpak et al [66] also found that their study sample of people with OUD engaged more with the peer navigator than the remote intensive outpatient program that the peer navigator was supporting. It is thus possible that this component of the intervention contributed significantly to the study findings of increased scores on perception of importance in change in substance use and the observed reduction in substance use. These study findings are supported by an extensive body of literature demonstrating the effectiveness of peer-based interventions in improving a range of outcomes related to SUD, including increased retention in SUD treatment and reduced rates of relapse [67]. The ability of peer navigators to engage with people who use drugs in culturally congruent ways, role model the substance use recovery journey, and provide a range of supports, including recovery and other social support (informational and emotional support), likely contributes to the effectiveness of peer recovery support interventions. The Chat feature and reminders within the mobile app could facilitate contact with peer navigators, potentially augmenting the positive effects of PRSS.

Overall, while nearly 3 quarters of participants who completed the 3-month follow-up reported the use of mobile app at least once, mobile app components of the intervention were used infrequently. The data on uptake of mobile app tools for substance use treatment or recovery support is mixed, with some studies reporting high uptake and effectiveness and others reporting low uptake [68,69]. Previously reported reasons for low uptake or effectiveness of mobile apps include low perceived usefulness, having limited access to or comfort with the technology, privacy concerns, and lack of personal motivation to engage in the intervention [68-71]. Similarly, we found that the reasons for not engaging with the app during the pilot period (ie, forgetting about the app, losing the phone, losing access to the app, and forgetting how to use the app) aligned with the ideas of low perceived usefulness or motivation and a lack of access to and comfort with the technology; though, interestingly, none of the participants in our study raised privacy concerns. The component of the mobile app that participants engaged with most frequently was the “Stories” section. The visual narratives may have thus contributed to the provision of information with the potential to change motivation and behavioral skills related to substance use recovery and engagement in care for OUD. Engagement with narratives may have been limited, with only 2 narratives embedded in the app.

The graphic narrative and animation formats were chosen with the aim of harnessing the ability of visuals to enhance learning [72-75] and the power of narrative transportation to affect behavior change [48-50] and appear to have been successful overall. Participants who engaged with the stories generally reported favorable responses related to transportation into the narratives (Table 5). Recipients with a lower self-reported ability to generate vivid mental imagery have been shown to experience lower levels of transportation into text-based narratives than narratives with accompanying visuals [76]. The graphic narratives and animations in this intervention may have assisted with overcoming this barrier by enabling such recipients to experience higher levels of transportation.

The development of narratives from remixes of reported lived experiences offered the opportunity to convey sensitive narratives about people with OUD visually. Sequential drawings, as opposed to fotonovelas [77] or video, allowed for depictions of characters in simplified, iconic visual form, which may differ from photographic representations in their ability to communicate information efficiently [78]. Visualizing topics, such as OUD-related injuries in an iconic style, also avoids photorealism of potentially disturbing content.

Video testimonials have been used as a means of persuading patients to taper off opioids for chronic pain [79]. A drawback of using video testimonials for OUD is the lack of storyteller anonymity required while discussing sensitive personal topics. Purely text-based testimonials, which would allow anonymity, may be less engaging for individuals with low literacy levels or low ability to generate vivid mental imagery [76]. The visual narratives allowed for anonymity of the storytellers, which enabled a vivid portrayal of content and behaviors that might not be discussed in testimonials. The format has potential as an effective approach to conveying health information related to SUD. Future studies could investigate the effectiveness and associated advantages and disadvantages of the visual narrative format and a peer-based format for narrative health communication.

### Limitations

While this study has several strengths, including a behavior therapy-driven and systematic approach to intervention development, incorporating the perspectives and experiences of people with OUD and care providers to people with OUD in intervention development, and successful nonincentivized intervention deployment, the study also has several limitations. These limitations include the observational nature of this pilot study, with only 20 participants enrolled. We are unable to demonstrate which components of the intervention (peer navigation or mobile app support components) were most effective. Until we can investigate further, it is our belief that both parts of the intervention were integral to driving the observed outcomes. We were also unable to capture metrics for mobile app usage and thus unable to quantitatively describe the use of mobile app components. Finally, of the 219 patients referred for study participation, 85 (38.8%) failed to meet eligibility criteria due to not possessing a smartphone. This represents a lower rate of smartphone ownership than in a recent study of 494 people who inject drugs in Fresno, CA, where 13% did not own a smartphone [80]. This exclusion criterion in this study could have led to participants having less severe OUD, given that people with OUD who have cellphones have been shown to be less likely to be unhoused, share syringes, and reuse syringes [80]. More severe OUD could also be associated with a higher likelihood of ED visits, leading to lower smartphone ownership in this sample. Future evaluations of the intervention with a randomized controlled design could include a printed version of the intervention’s content or include playing the animated versions of the narratives in the hospital to broaden participation to people with OUD without smartphones.

### Conclusions

This study demonstrates the feasibility of the development and deployment of a peer-led, narrative-based intervention to support SUD care engagement and preliminary data in support of acceptability, appropriateness, and effectiveness. These data show a reduction in substance use at 3-month follow-up compared to baseline. The embedded visual narratives represent a promising approach for provisioning information and potentially evoking behavior change in accordance with the IMB model and transportation theory. Rigorous evaluation with a randomized controlled design in a larger population is needed to determine intervention effectiveness and which components of the intervention are critical for effectiveness.

## Supplementary material

10.2196/82485 Multimedia Appendix 1 Messaging and experiences for narratives mapped to the transtheoretical model.

10.2196/82485 Multimedia Appendix 2 Pros and cons of entering treatment for opioid use disorder and additional factors affecting treatment success.

10.2196/82485 Multimedia Appendix 3 Graphic narrative of one of the stories: Eva’s narrative.

10.2196/82485 Multimedia Appendix 4 Graphic narrative of one of the stories: James’s narrative.

10.2196/82485 Multimedia Appendix 5 Full animation of one of the stories: Eva’s narrative.

10.2196/82485 Multimedia Appendix 6 Full animation of one of the stories: James’s narrative.

10.2196/82485 Multimedia Appendix 7 Fisher tests for app use and peer navigator engagement and the associations with opioid use and MOUD engagement. MOUD: medication for opioid use disorder.

## Abbreviations

AIM: Acceptability of Intervention Measure
ED: emergency department
IAM: Intervention Appropriateness Measure
IMB: information-motivation-behavioral skills
MAUQ: mHealth App Usability Questionnaire
mHealth: mobile health
MOUD: medication for opioid use disorder
OUD: opioid use disorder
PRSS: peer recovery support services
SUD: substance use disorder
TCU: Texas Christian University
TS–SF: Transportation Scale–Short Form
TTM: transtheoretical model
TUQ: Telehealth Usability Questionnaire
USE: Usefulness, Satisfaction, and Ease of Use
