# Supplementary material for: A Peer-Led, Narrative-Based, and Mobile-Supported Intervention in Opioid Use Disorder: Multiphase Qualitative and Longitudinal Observational Study
Source: JMIR Form Res. 2026 Feb 5;10:e82485. doi: 10.2196/82485 (PMC12875427; doi:10.2196/82485)
Supplement: Multimedia Appendix 1 [file formative-v10-e82485-s001.pdf]

Supplemental Material © 2025 JHU, from Latimer MC, Gregg L, Saheed M, Tobin KE, Kelly SM, Agee T, Steele RJ, Frankiewicz N, Verinumbe T, Latkin C, Falade-Nwulia O. *A Peer-Led, Narrative-Based, and Mobile-Supported Intervention in Opioid Use Disorder: Formative and Pilot Research*. JMIR Formative Research

	Precontemplation	Contemplation	Preparation	Action	Maintenance (Relapse, stability)
Stage	-No intention to take action in the next 6 months<br>-Unaware behavior is cause of negative consequences<br>-Underestimates the positives of making a change	-Recognizes behavior is a problem<br>-Ambivalent about changing<br>-Pros of change seem equal the cons	-Ready for action in the next 30 days<br>-Taking small steps to change<br>-Believes change will lead to positive outcomes	-Taking steps to change<br>-Changes made for less than 6 months	-Changes made for more than 6 months<br>- May include relapse and return to Maintenance with potential to reach a strong sense of stability
Messages and experiences/ events (“exemplars”) associated with each stage	• Sees people die of causes related to opioid use disorder (OUD)<br>• Damages overall health<br>• Experiences a life-threatening event with hospitalization<br>• Told they will die if you don’t stop using drugs<br>• Damages done to family relationships<br>• Loss of employment<br>• Experiences financial barriers created by OUD<br>• Experiences difficulty treating other health problems<br>• Needs drugs in order to feel normal<br>• Loss of belongings<br>• Exposure to dangerous situations, assaults<br>• Exposure to the cold<br>• Experiences food instability<br>• Feels sick every day<br>• Spends every day looking for drugs<br>• Shows inability to support family emotionally and financially<br>• Emotionally disorganized<br>• Has limited access to medication-assisted treatment (MAT)	• Discusses applicable pros and cons with Peer Mentor<br>• Addresses skepticism of treatment due to bad prior experience<br>• Addresses misinformation<br>• Destigmatizes OUD as a medical, not ethical, problem.<br>• Understands medication can relieve the pain of withdrawal<br>• Addresses fear of discovering new health problems while in drug treatment<br>• Addresses desire to maintain relationships with others that use<br>• Understands treatment is not as punitive as it used to be, it’s about harm reduction.<br>• Receives information on OUD, basic neuroscience of how MAT works<br>• Understands MAT is not “just trading one drug for another”	• Ends an abusive relationship<br>• If residential treatment, prepares to enter facility<br>• Cuts down on use<br>• Sets clear goals for action<br>• Overcomes difficulties navigating healthcare system<br>• Learns what to expect when in drug treatment<br>• Arranges support for initiation of Suboxone<br>• Distances self from friends and acquaintances that use drugs<br>• Learns how to start MAT without inducing precipitated withdrawal.	• Experiences that withdrawal was not as bad as they thought.<br>• Understands cravings for opioids are managed on the right dose.<br>• Experiences good treatment and support<br>• Continues to isolate from friends and acquaintances that use drugs<br>• Initially skeptical of group support<br>• Learns from others in recovery<br>• Learns to control and avoid triggers<br>• Overcomes the boredom of not using and learns to be themselves<br>• Learns why relapse can be dangerous<br>• Learns that people don’t ridicule you when you tell your story in groups<br>• Understands treatment must be a “selfish” program in order to work<br>• Learns to put own well-being first<br>• If in residential treatment, adjusts to being near other patients<br>• Avoids entering a relationship during early phases of treatment with someone who is also beginning treatment.<br>• Builds coping skills, your “toolbox.”	• Mind clears, experiences feeling well, and knowing how to engage in support.<br>• Takes responsibility for commitments<br>• Reestablishes family relationships and faces hurt loved ones, has to apologize<br>• Maintains correct dosage of medications<br>• Understands recovery is a lifetime of work<br>• Addresses goals one step at a time<br>• Experiences a troubling situation that leads to relapse: abusive relationship, family death, illness, depression, or anxiety<br>• Experiences Peer Mentor encouragement<br>• Is goal-oriented instead of focusing on mistakes.<br>• Forgives themselves and moves on after a relapse.<br>• Associates with others who are committed to recovery.<br>• Participates in a good support group<br>• Shares stories as a reminder of how far they have come.<br>• Controls and avoids relapse triggers, access to drugs.<br>• Manages feelings of isolation<br>• Experiences stable family relationships<br>• Has confidence in their ability to stay in recovery<br>• Works through new health problems<br>• Re-enters a community environment after residential treatment.

Supplement 1. Narrative messaging and experiences mapped onto the transtheoretical model (TTM). The TTM provided a foundation for organizing narrative elements around the stages of change (row 1). Narrative elements were derived from the pros and cons, barriers to change, attitudes and beliefs, and information needed to pursue treatment documented in Supplement 2.
