# Supplementary material for: A Peer-Led, Narrative-Based, and Mobile-Supported Intervention in Opioid Use Disorder: Multiphase Qualitative and Longitudinal Observational Study
Source: JMIR Form Res. 2026 Feb 5;10:e82485. doi: 10.2196/82485 (PMC12875427; doi:10.2196/82485)
Supplement: Multimedia Appendix 2 [file formative-v10-e82485-s002.pdf]

Pros of entering treatment for opioid use disorder

- Stop damaging personal health.
- Stop feeling ill every day and needing drugs to feel normal.
- Stop damaging relationships with family and friends (reduce feelings of isolation).
- Stop spending every day searching for drugs.
- Be able to successfully treat other health problems.
- Be able to keep a job.
- Be able to access to stable housing.
- Avoid overdosing.
- Lessen financial barriers created by OUD, be able to assist family members financially.
- Avoiding future cravings with MAT.
- Be able to think clearly and make decisions.
- Stop experiencing food instability.
- If living unhoused, stop living in dangerous situations and sleeping in the cold outside.

Cons of entering treatment for opioid use disorder

- Finding out about undiagnosed health conditions while in drug treatment.
- Illness during withdrawal.
- Risk of being treated poorly by treatment programs.
- Facing hurt loved ones, having to apologize.
- Facing the difficulty of ending abusive relationships.
- Needing to isolate oneself from all acquaintances and friends that still do drugs.
- Potentially needing to enter an in-patient facility.
- The possibility of ending up in a poorly run treatment program.
- Needing to travel to treatment and group meetings.
- Disliking support group discussions

Barriers to change

- Access to healthcare
- Difficulties navigating the healthcare system even with insurance, Medicare or Medicaid.
- Transportation: physical distance of program facilities, appointments, and meetings.
- Abusive relationships, family abuse situations.
- Family members and loved ones with OUD.
- Exposure to triggers and continued easy access to drugs (e.g. living near a seller).
- Re-entering a community environment after residential treatment.
- Healthcare system:
  - Quality of treatment from program to program differs substantially.
  - Insurance with limited coverage.
  - Difficulties navigating a complex system:
    - Arduous intake processes for programs.
    - Interpersonal communication and organizational skills being affected by OUD and other conditions.
    - No linkage: patients lose contact during the transition to treatment.
  - Care provider actions:
    - Demanding abstinence: some patients just want to stay alive rather than completely stop drugs.
    - Shaming and taunting patients for not adhering to treatment.
    - Inciting fear of being kicked out of a program for not adhering.

Attitudes and beliefs

- Stigmatization of addiction as an ethical instead of medical problem.
- Fear of the unknown within treatment programs causing extreme anxiety.
- Bad prior experience leading to skepticism of OUD treatment.
- Misinformation:
  - Methadone rots teeth.
  - Medication-assisted treatment (MAT) doesn't work.
  - Being on MAT means you're not "clean".
  - Minimizing dangers of not treating OUD.
  - Abstinence is required to be all programs.

Information needed to pursue treatment

- How to use Narcan during an OD.
- How to start MAT without inducing precipitated withdrawal.
- How to develop coping skills.
- Basics of opioid use neuroscience. Comparison of effects of heroine to suboxone.
- How to access treatment programs.

Supplement 2. Pros and Cons of entering treatment for opioid use disorder and additional factors affecting treatment success. During the design of the narratives, factors that were identified by interview participants as being foundational to the decision to seek or not seek treatment for OUD were organized into pros and cons and documented alongside factors affecting an individual's ability to remain in recovery including attitudes and beliefs, barriers to change, and information needed to pursue treatment.
