# Supplementary material for: A Peer-Led, Narrative-Based, and Mobile-Supported Intervention in Opioid Use Disorder: Multiphase Qualitative and Longitudinal Observational Study
Source: JMIR Form Res. 2026 Feb 5;10:e82485. doi: 10.2196/82485 (PMC12875427; doi:10.2196/82485)
Supplement: Multimedia Appendix 3 [file formative-v10-e82485-s003.pdf]

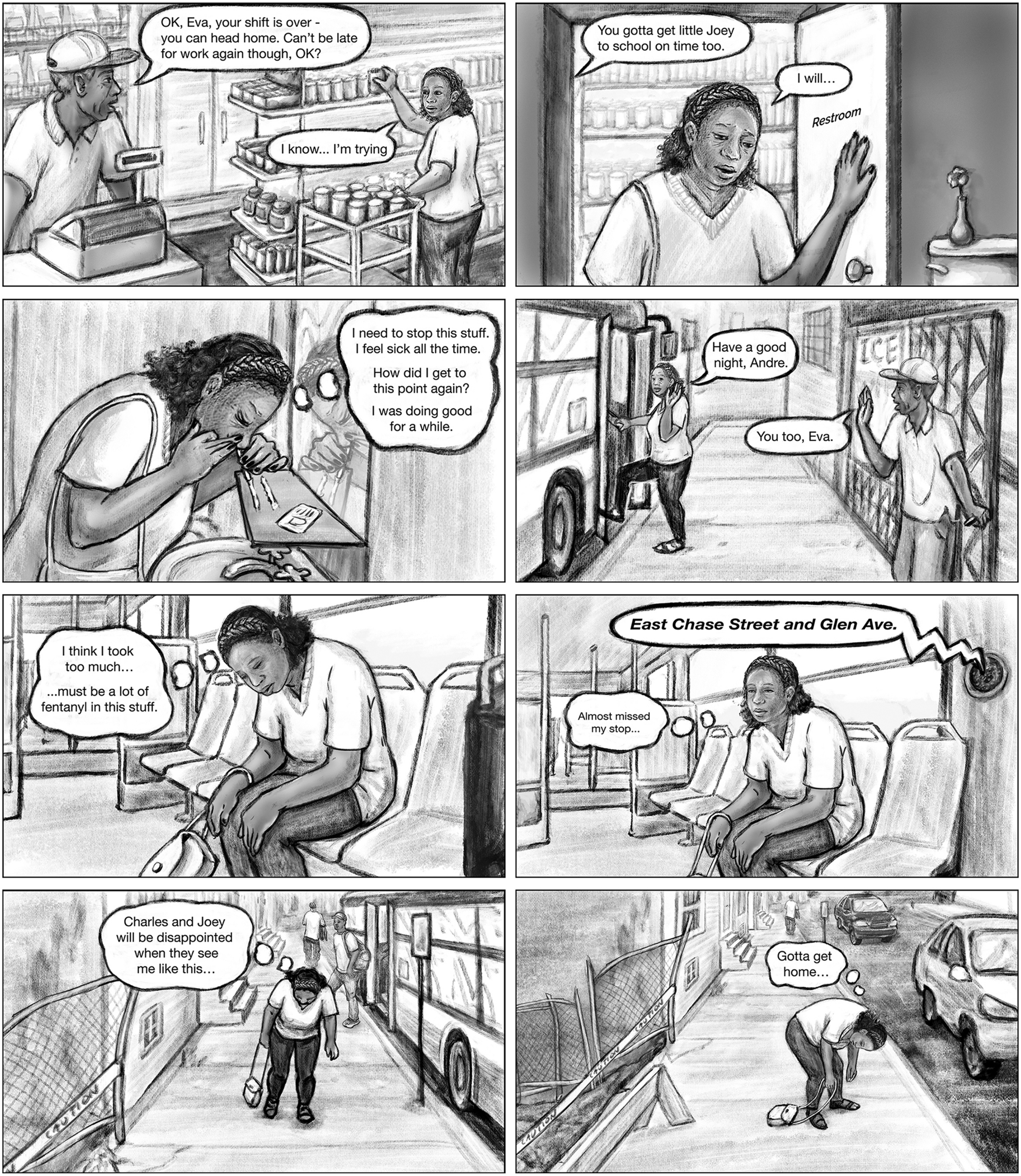

Images © 2022 JHU, illustrated by Lydia Gregg MA, CMI, FAMI

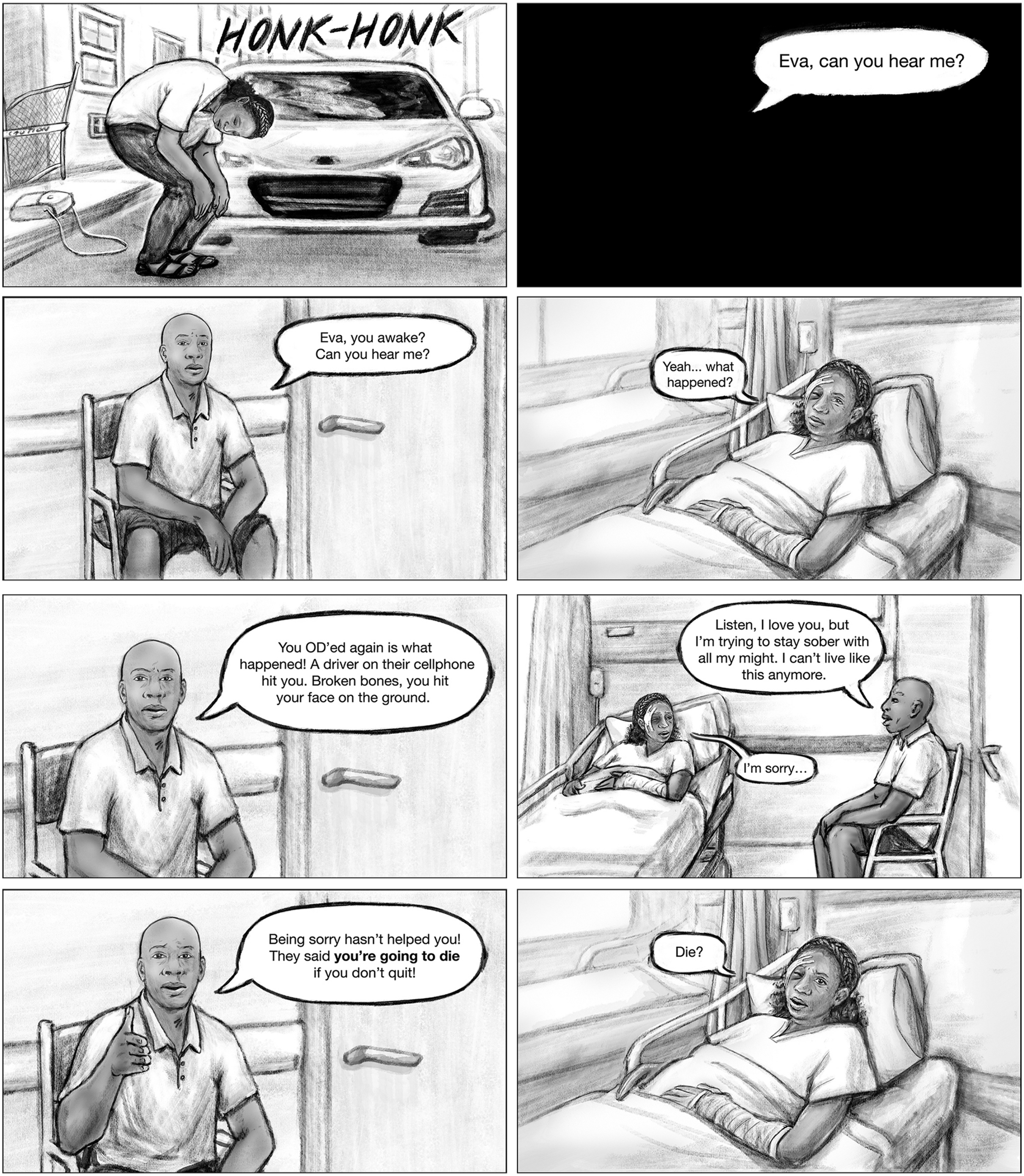

Images © 2022 JHU, illustrated by Lydia Gregg MA, CMI, FAMI

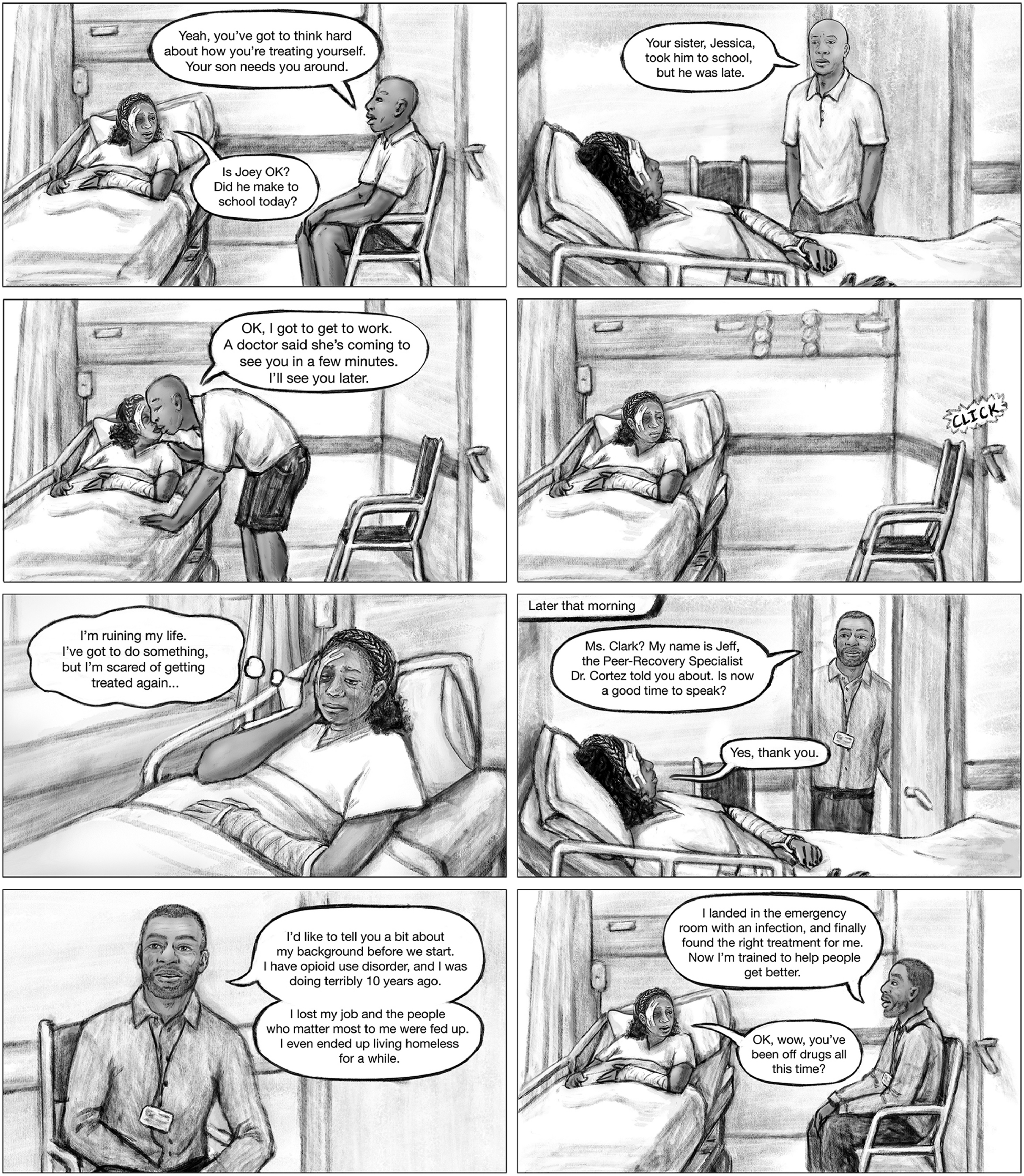

Images © 2022 JHU, illustrated by Lydia Gregg MA, CMI, FAMI

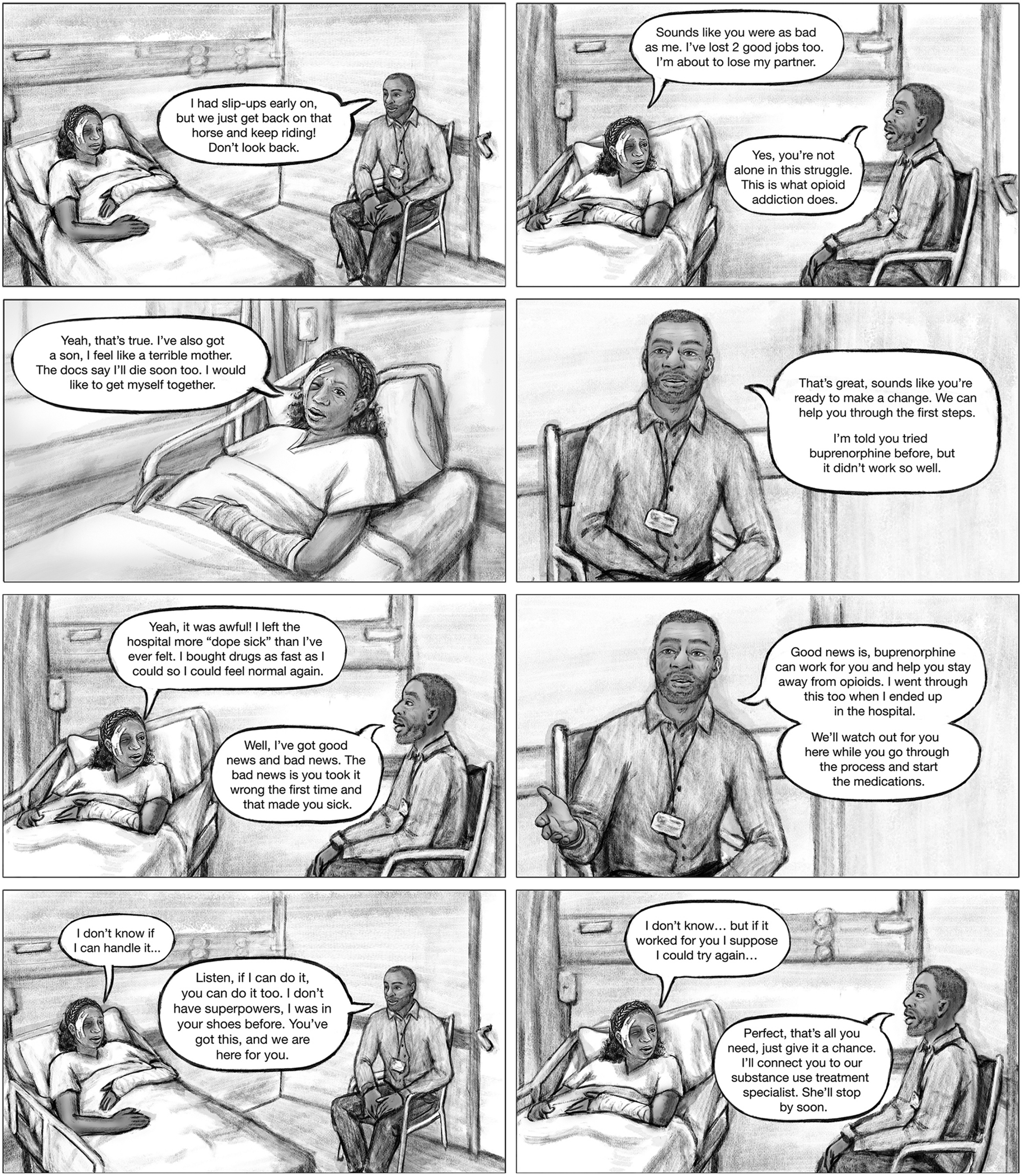

Images © 2022 JHU, illustrated by Lydia Gregg MA, CMI, FAMI

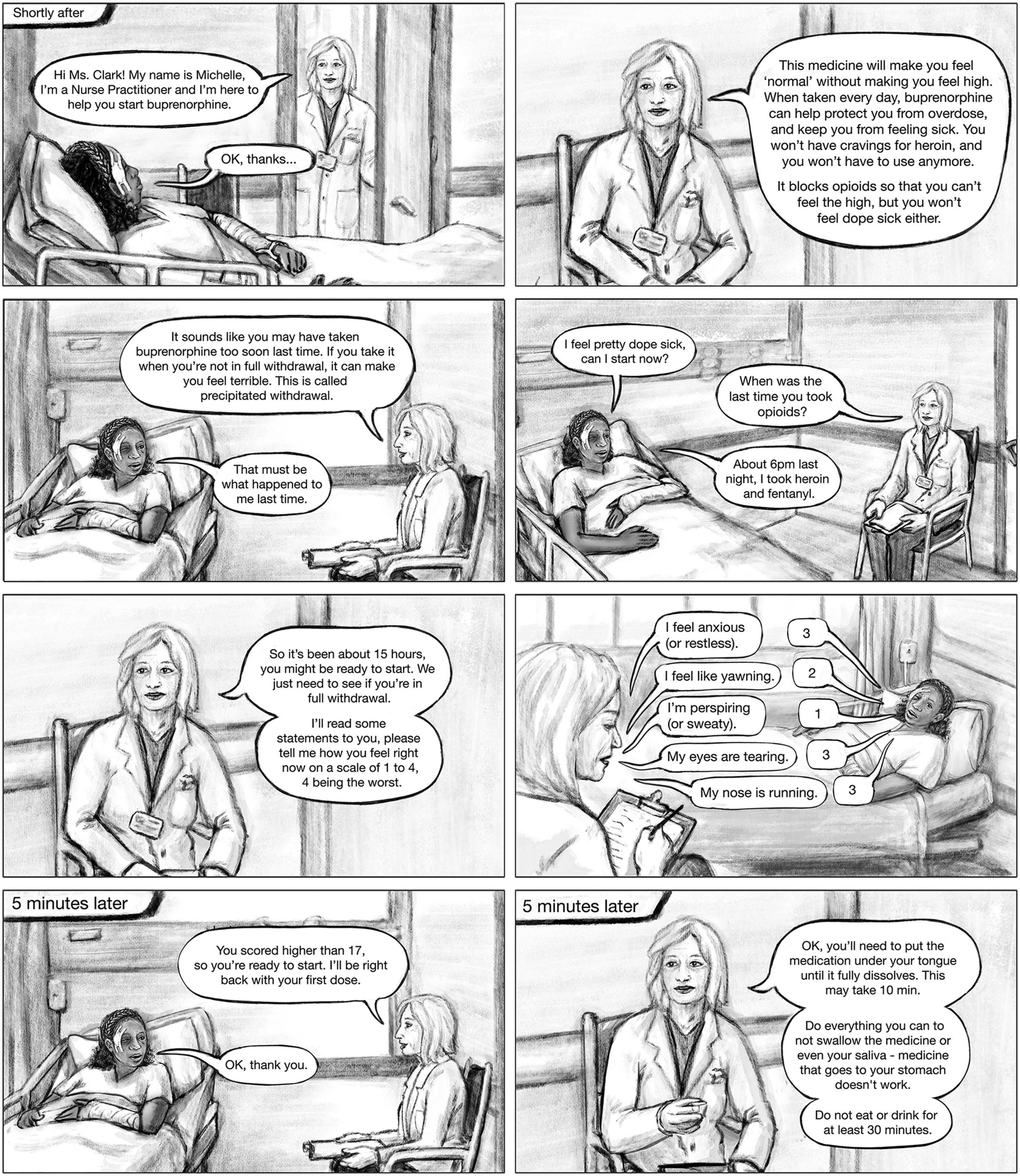

Images © 2022 JHU, illustrated by Lydia Gregg MA, CMI, FAMI

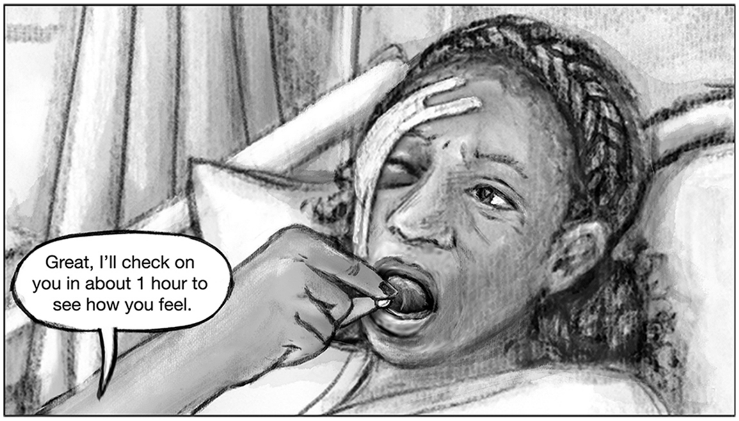

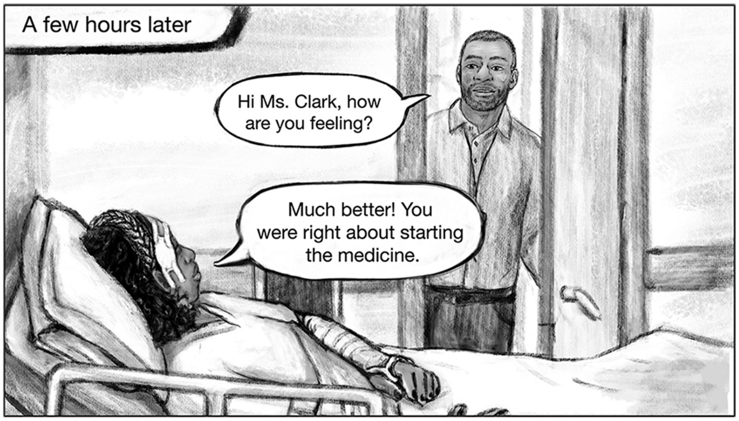

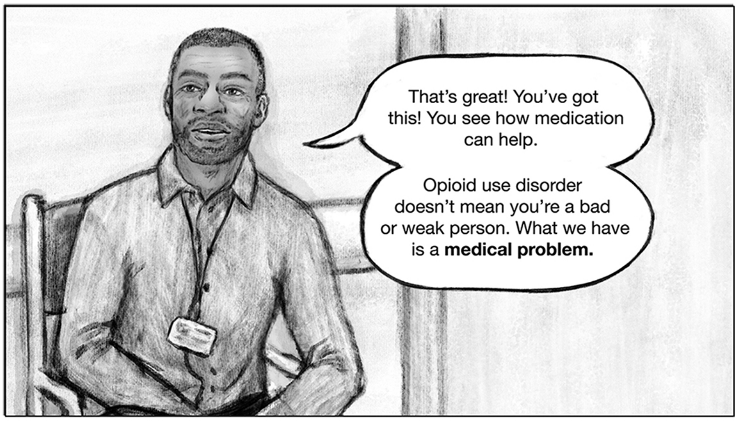

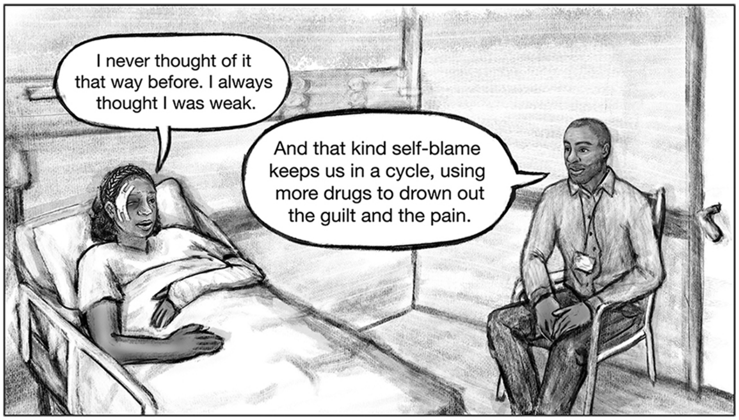

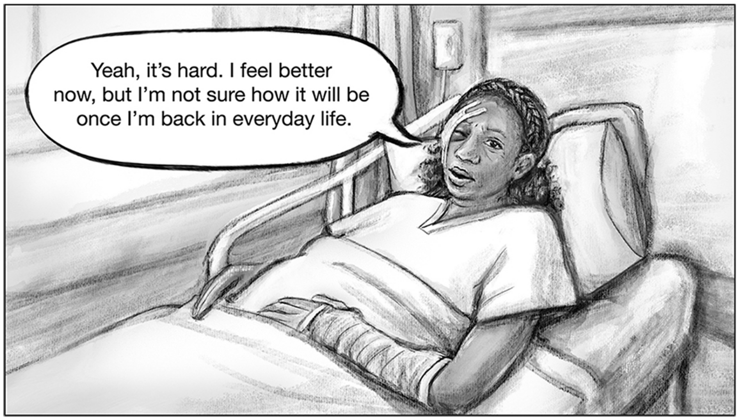

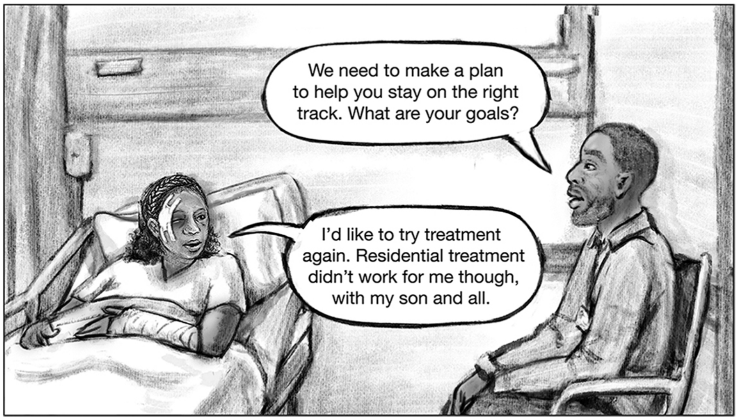

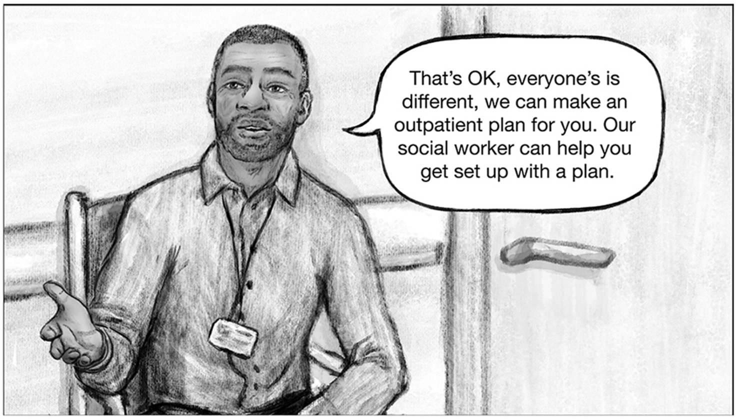

Eva's plan:

- Continue taking buprenorphine to treat her OUD.
- A hospital social worker and her Peer-Recovery Specialist will connect her to an outpatient program.
- Start therapy groups and Narcotics Anonymous (NA) meetings, and connect with a sponsor.
- Start family therapy with her partner and kids.
- Check-in with her Peer-Recovery Specialist (weekly)
- Keep a supply of Narcan in an easily accessible location, and always carry some with you.

Images © 2022 JHU, illustrated by Lydia Gregg MA, CMI, FAMI

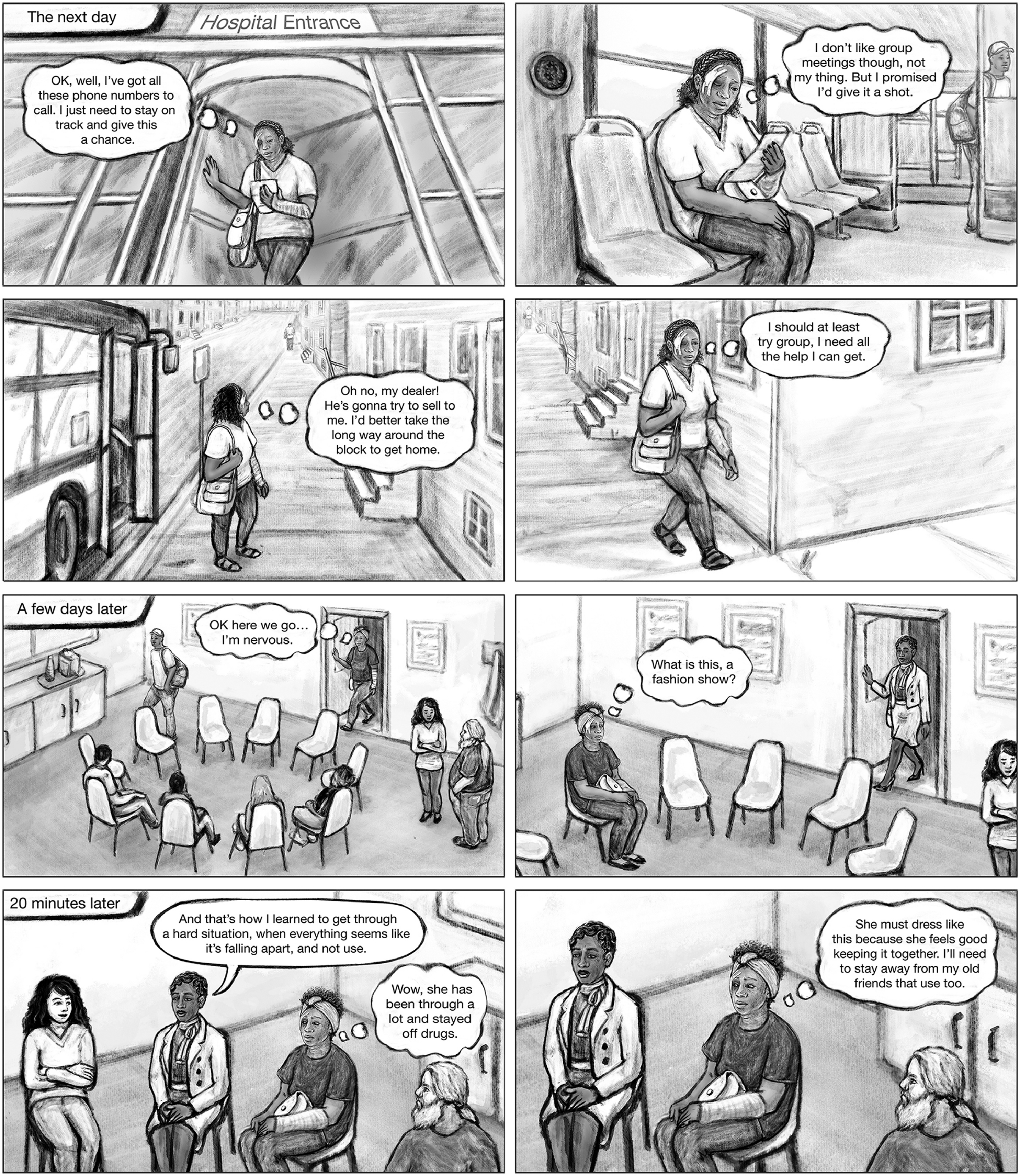

Images © 2022 JHU, illustrated by Lydia Gregg MA, CMI, FAMI

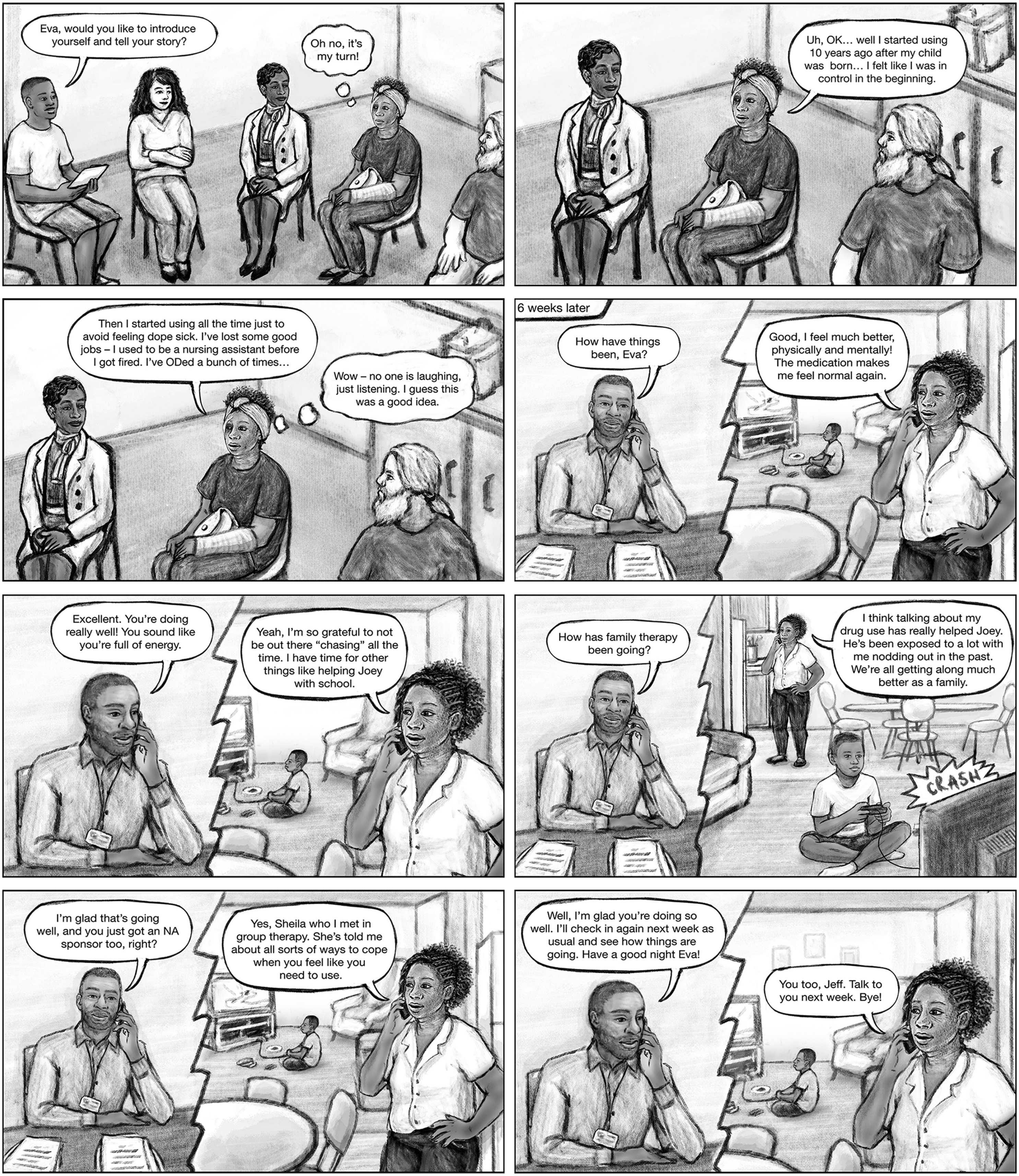

Images © 2022 JHU, illustrated by Lydia Gregg MA, CMI, FAMI

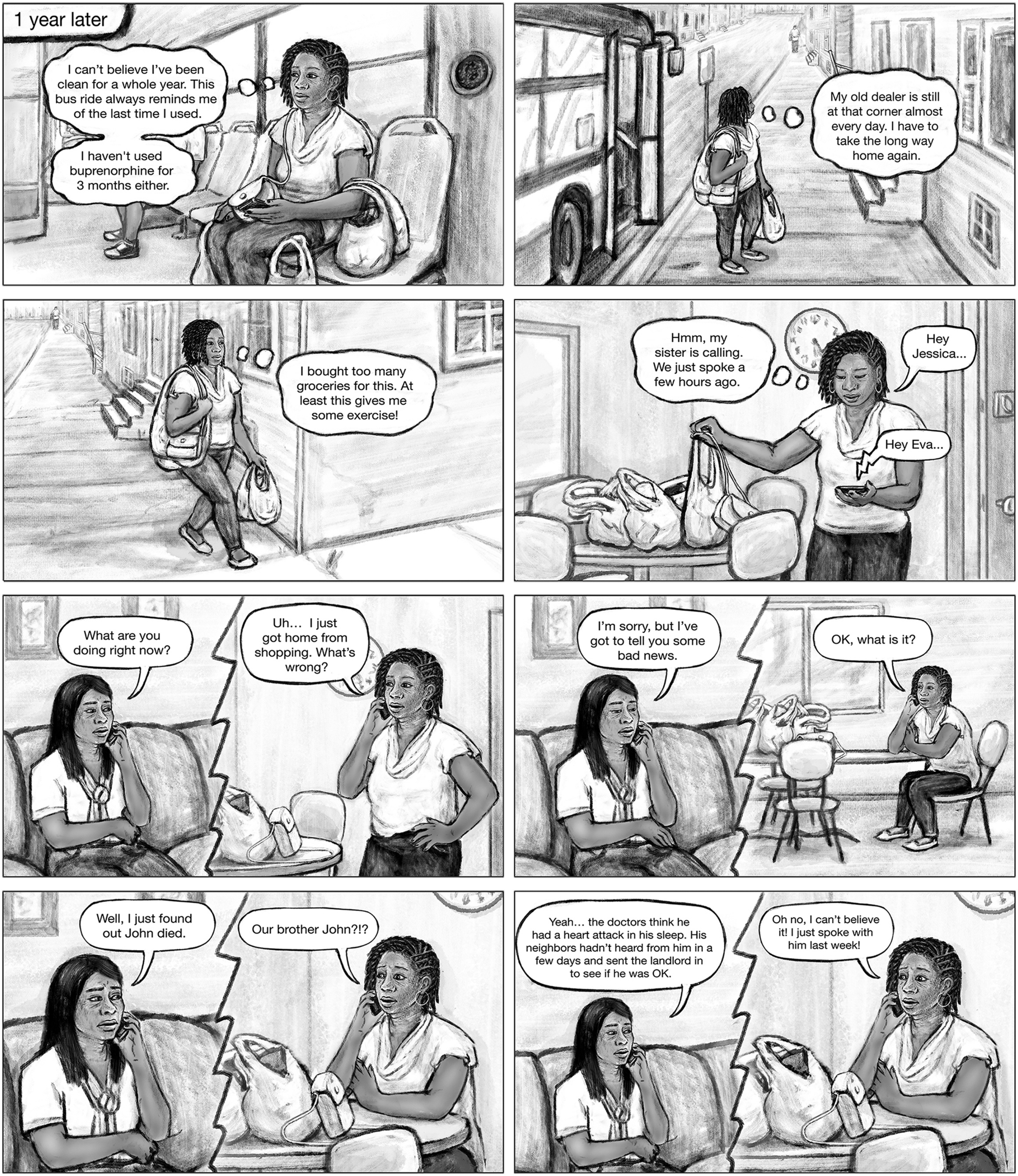

Images © 2022 JHU, illustrated by Lydia Gregg MA, CMI, FAMI

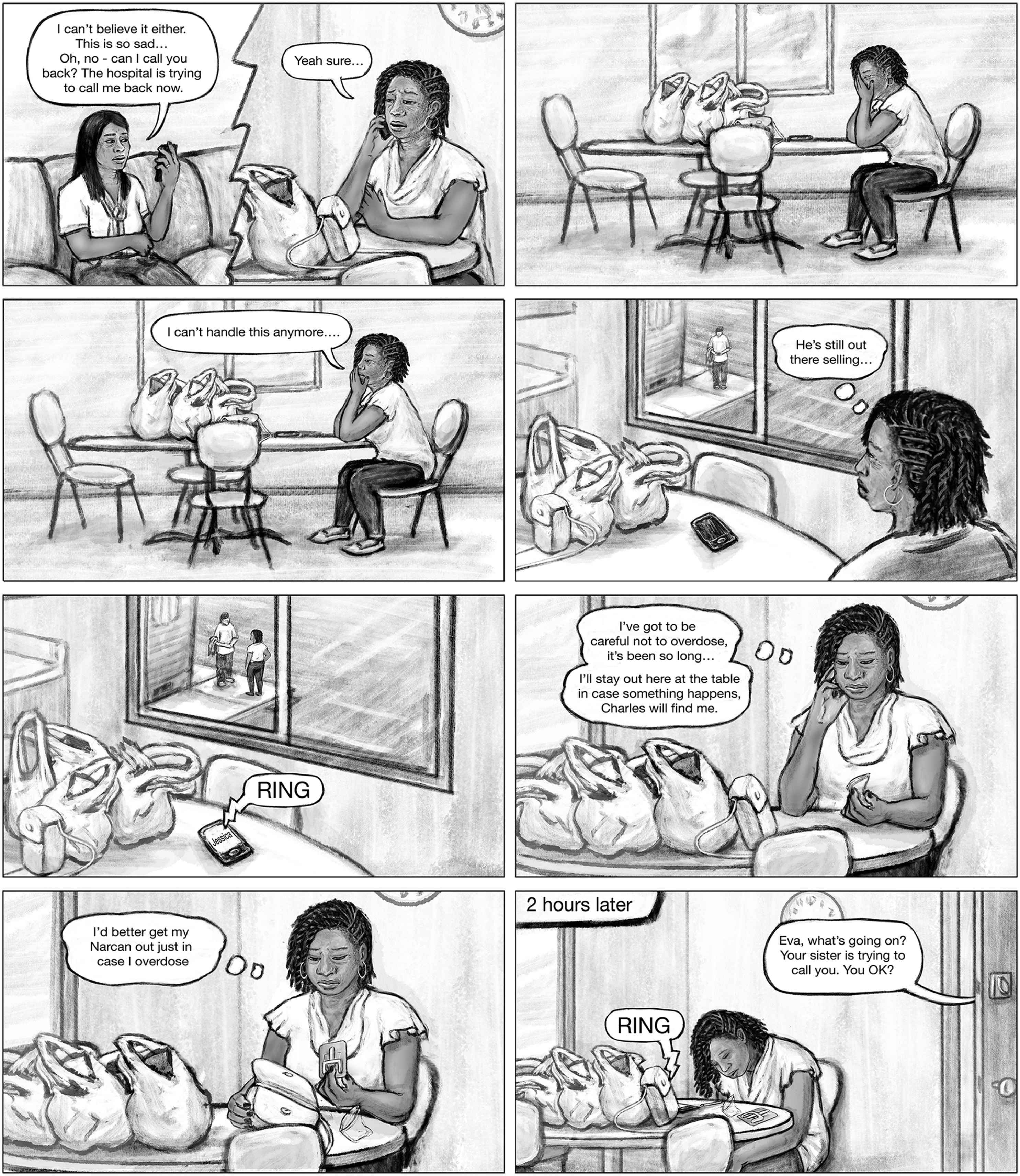

Images © 2022 JHU, illustrated by Lydia Gregg MA, CMI, FAMI

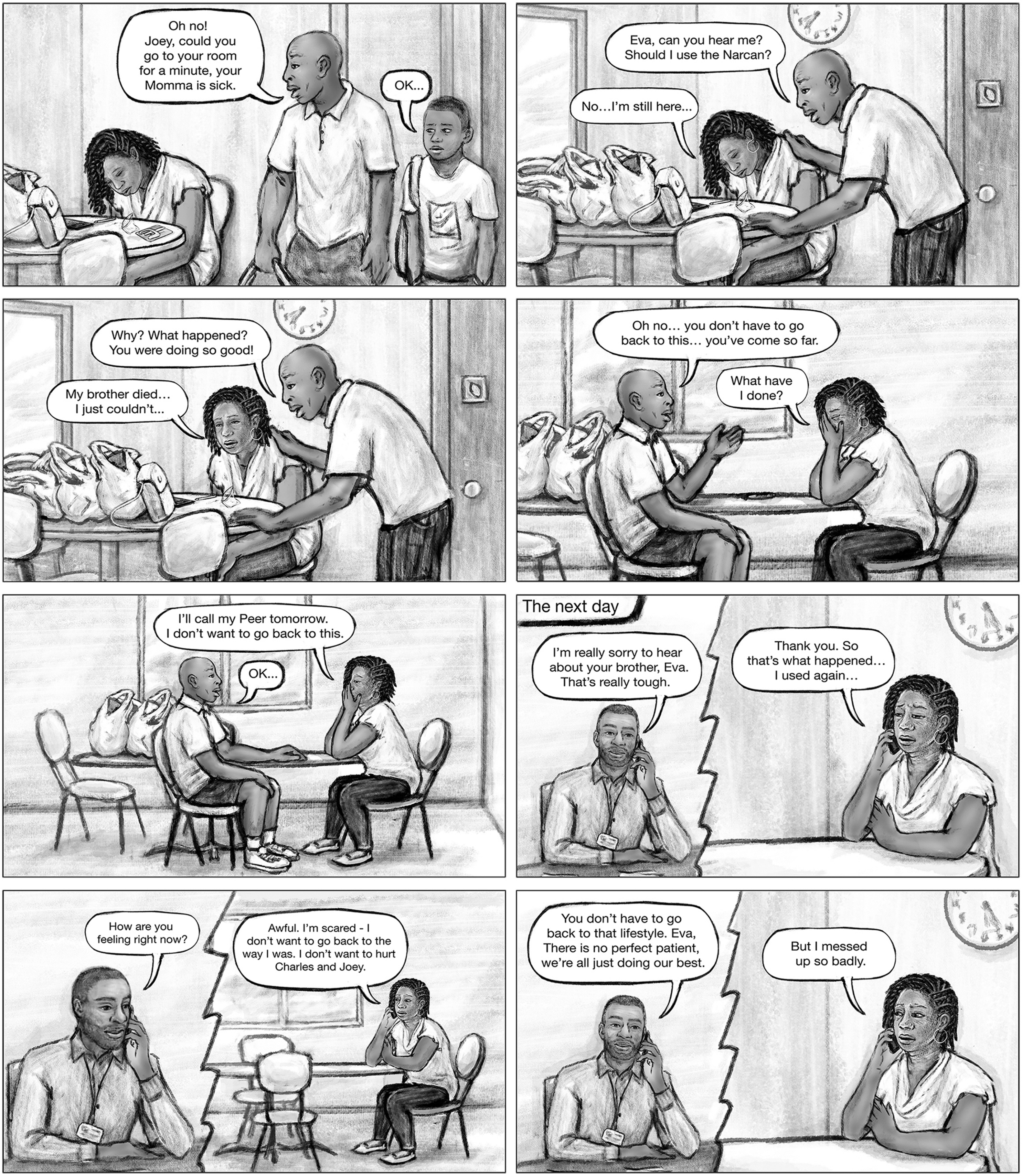

Images © 2022 JHU, illustrated by Lydia Gregg MA, CMI, FAMI

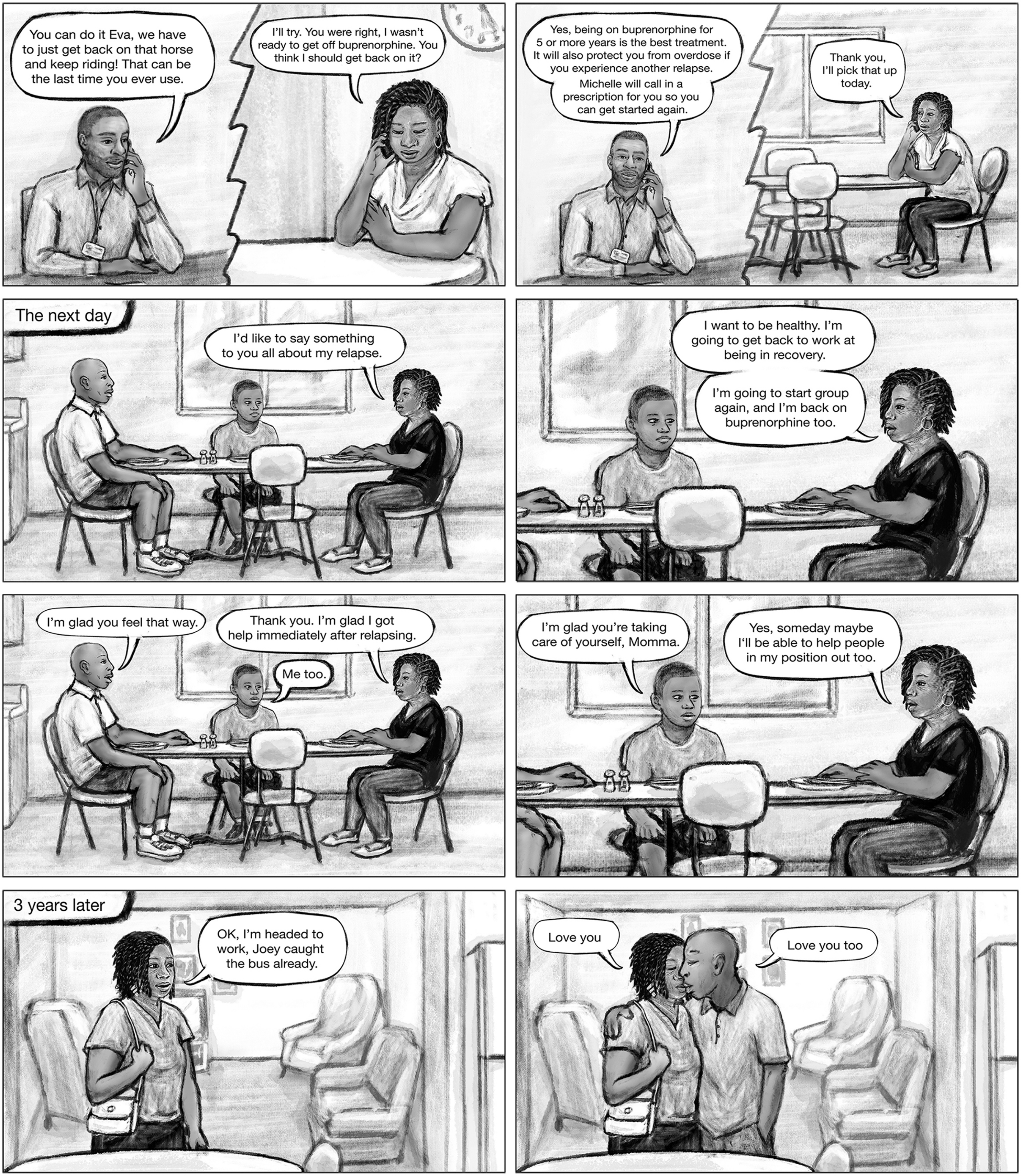

Images © 2022 JHU, illustrated by Lydia Gregg MA, CMI, FAMI

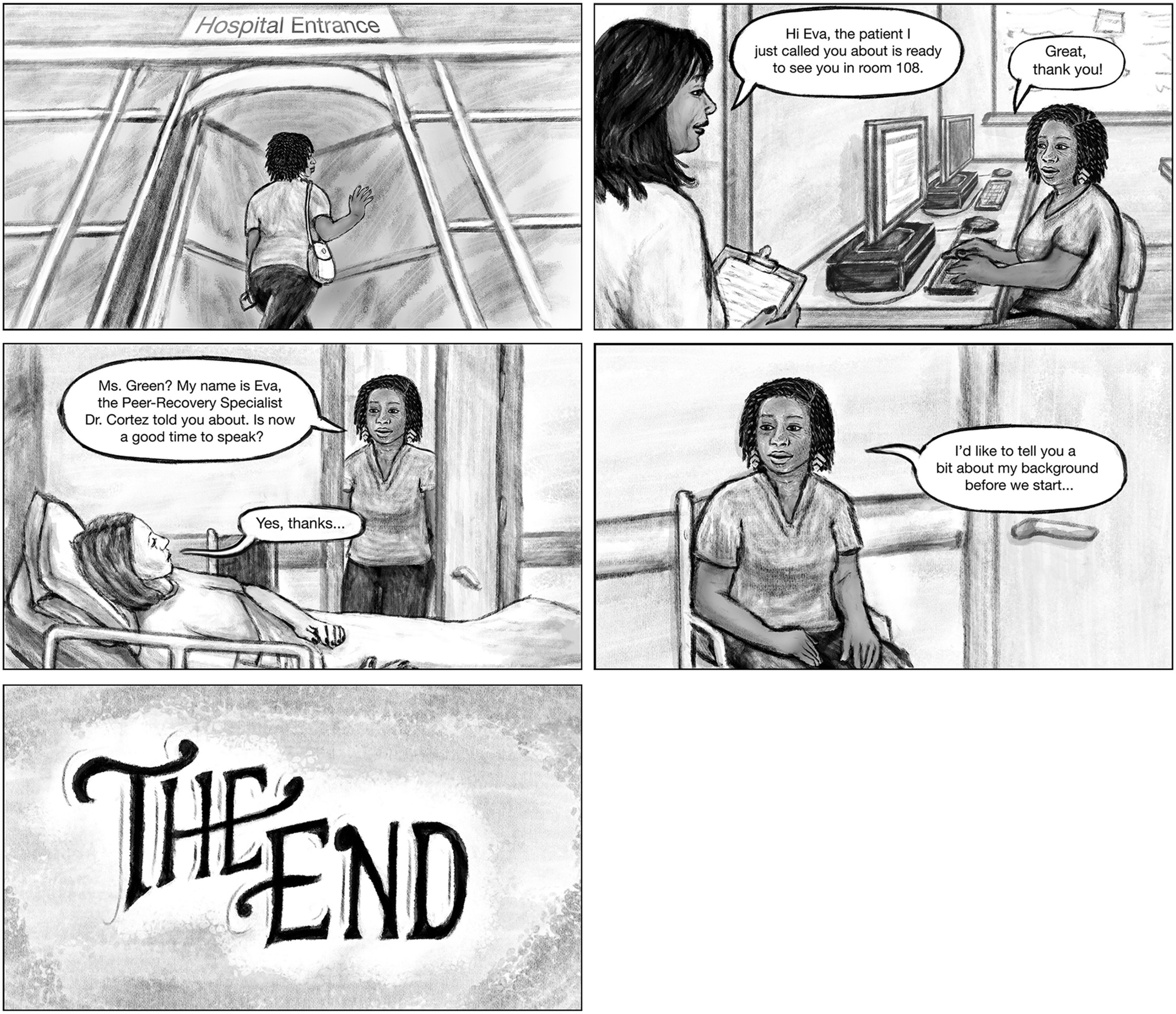

Images © 2022 JHU, illustrated by Lydia Gregg MA, CMI, FAMI
