# Supplementary material for: A Peer-Led, Narrative-Based, and Mobile-Supported Intervention in Opioid Use Disorder: Multiphase Qualitative and Longitudinal Observational Study
Source: JMIR Form Res. 2026 Feb 5;10:e82485. doi: 10.2196/82485 (PMC12875427; doi:10.2196/82485)
Supplement: Multimedia Appendix 4 [file formative-v10-e82485-s004.pdf]

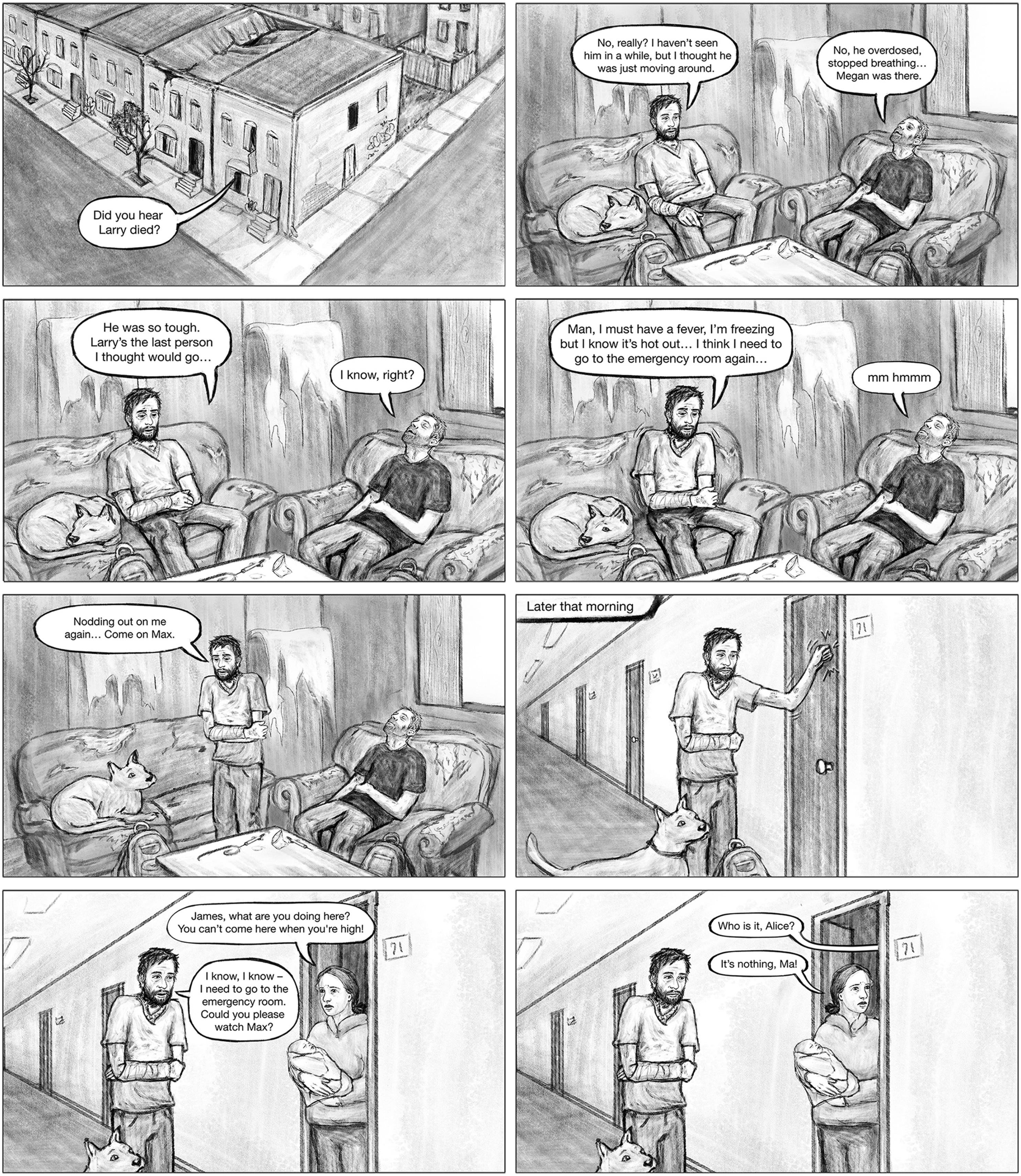

Images © 2022 JHU, illustrated by Lydia Gregg MA, CMI, FAMI

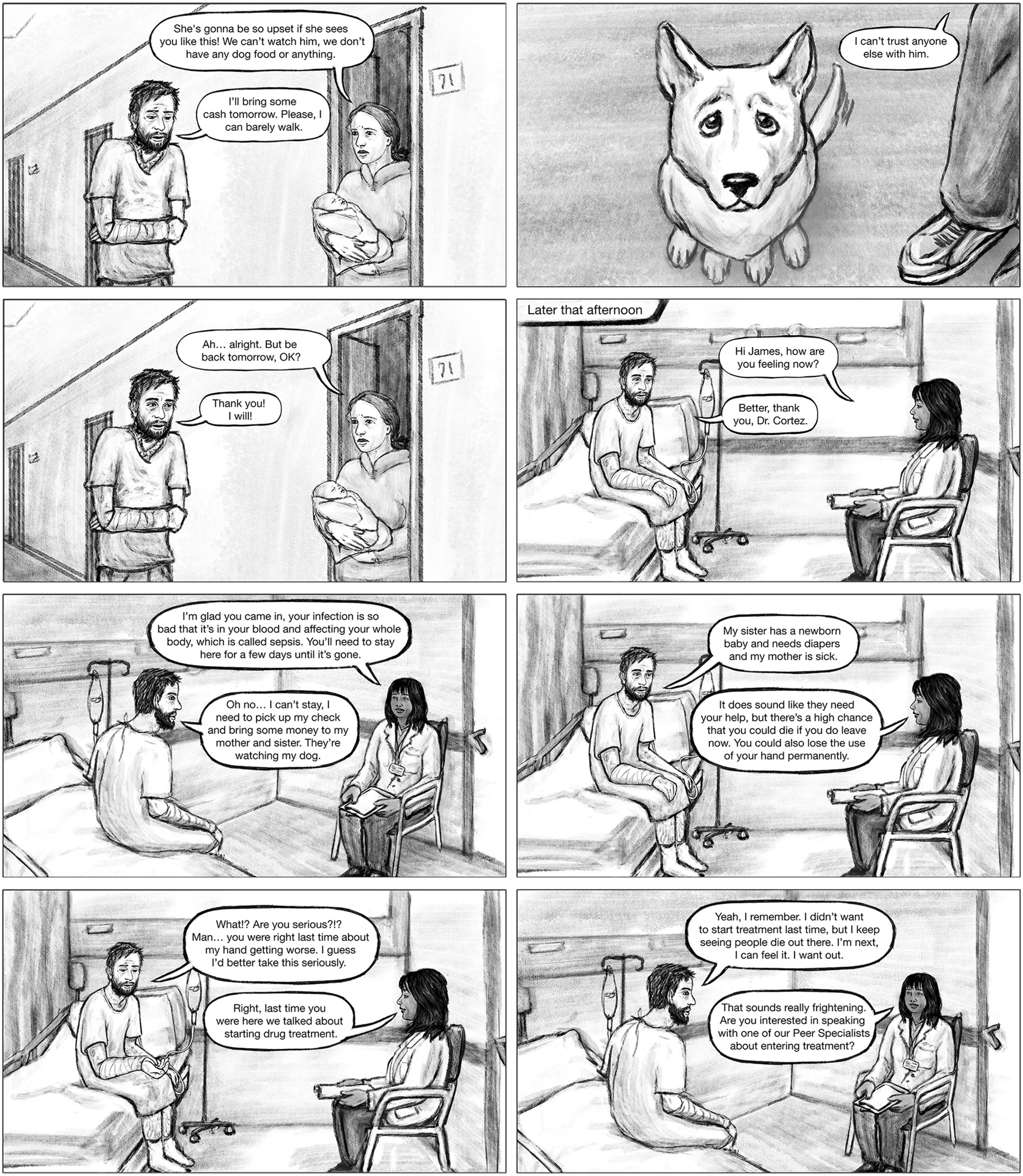

Images © 2022 JHU, illustrated by Lydia Gregg MA, CMI, FAMI

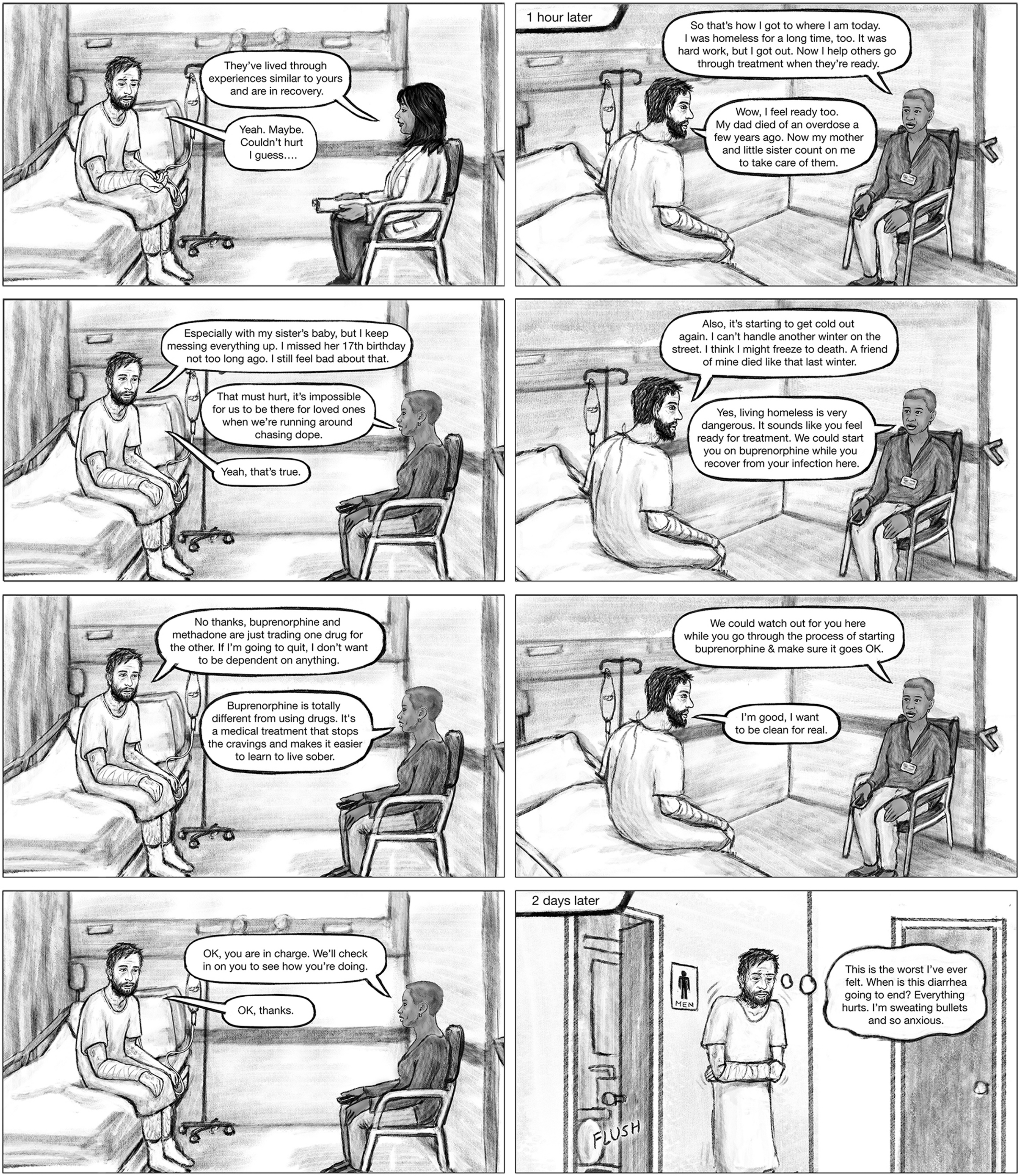

Images © 2022 JHU, illustrated by Lydia Gregg MA, CMI, FAMI

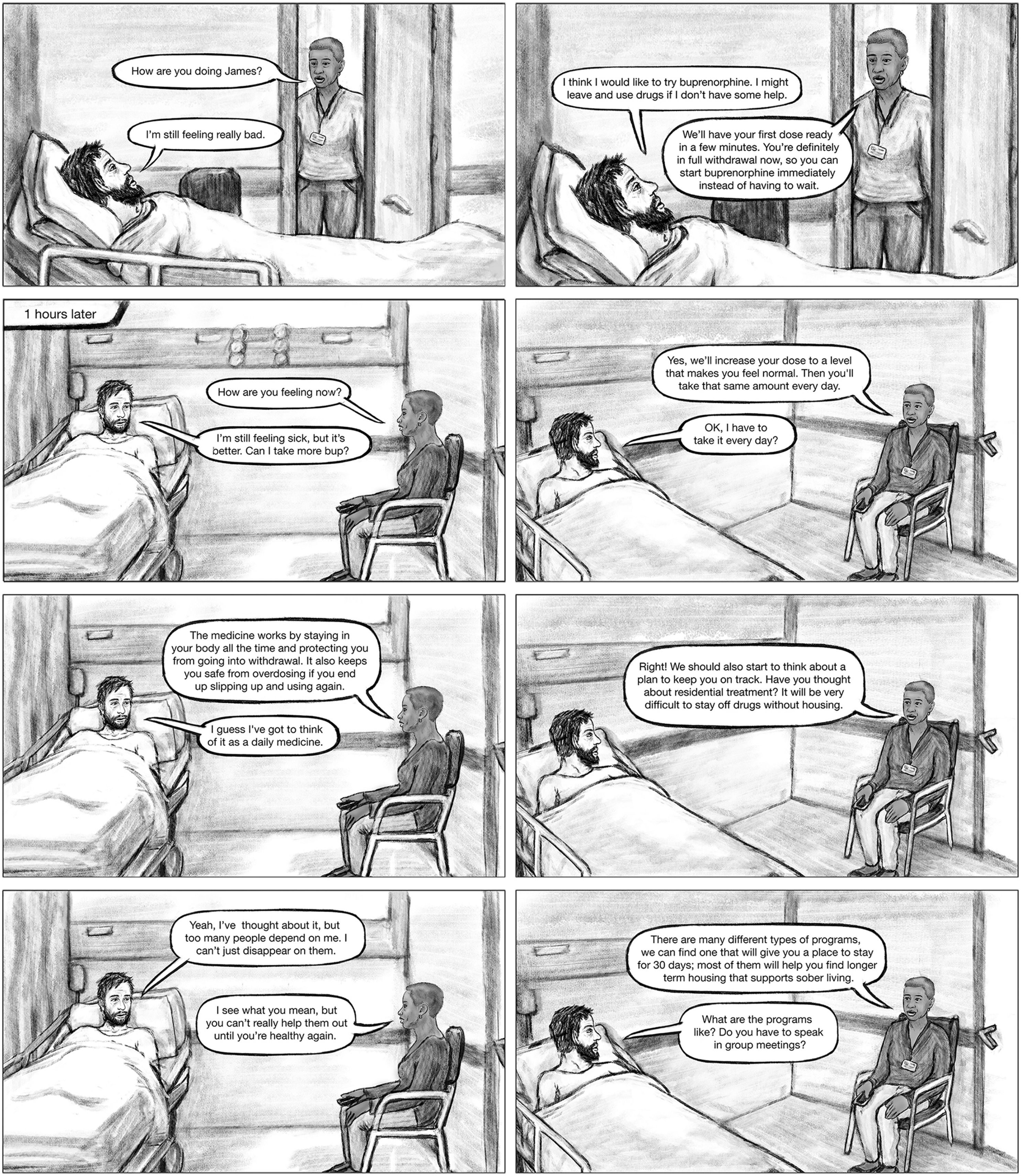

Images © 2022 JHU, illustrated by Lydia Gregg MA, CMI, FAMI

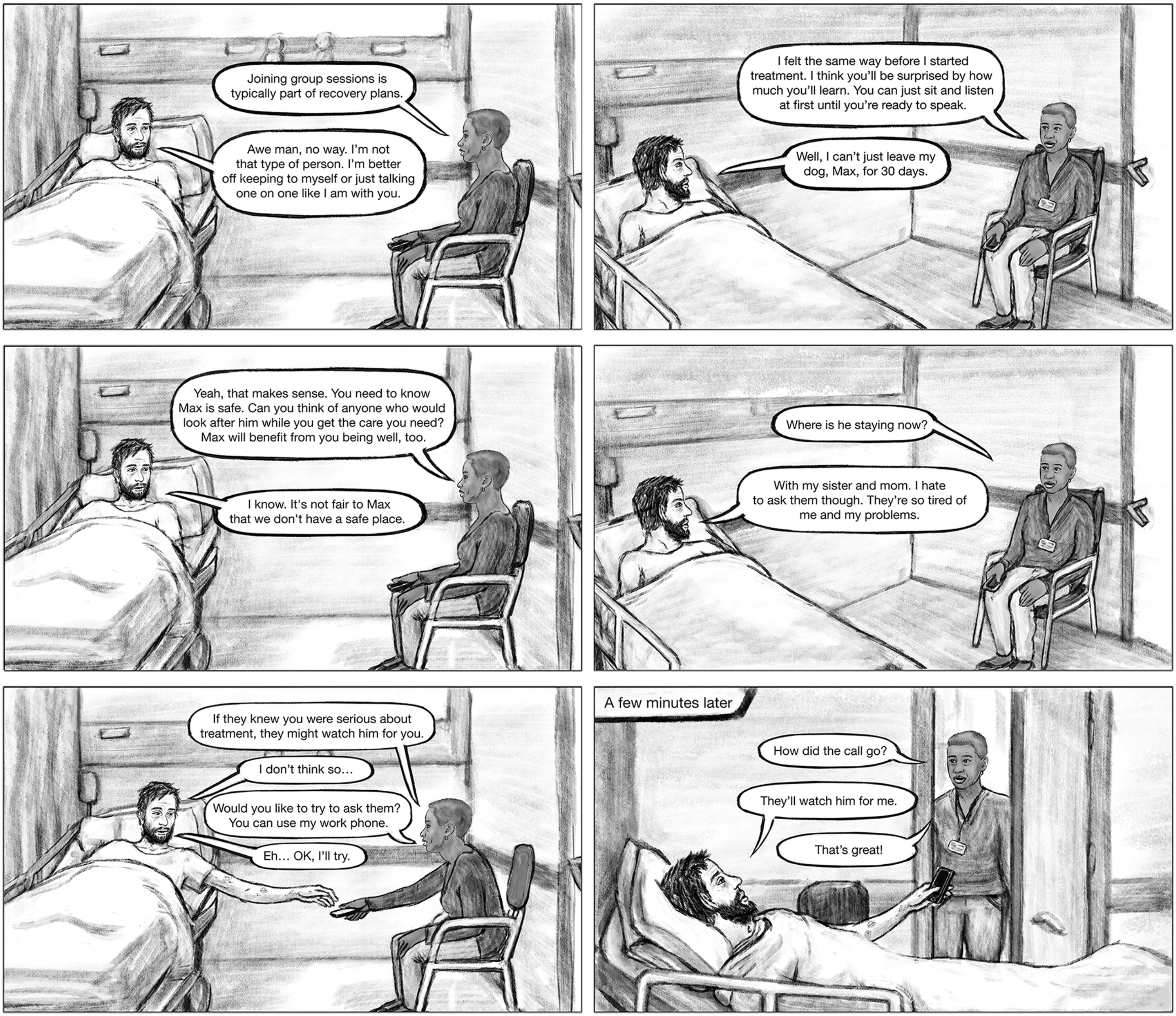

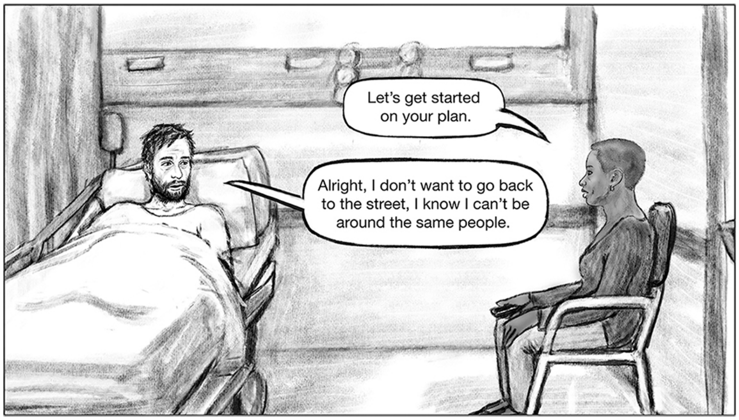

James' plan:

- Take buprenorphine as medicine every day.
- A hospital social worker and Peer-Recovery Specialist will connect him to a residential treatment program.
- Find a place to stay at a Sober Living house after treatment.
- Start therapy groups and Narcotics Anonymous (NA) meetings, and connect with a sponsor.
- Check-in with his Peer-Recovery Specialist (weekly).
- Keep a supply of Narcan in an easily accessible location, and always carry some with him.

Images © 2022 JHU, illustrated by Lydia Gregg MA, CMI, FAMI

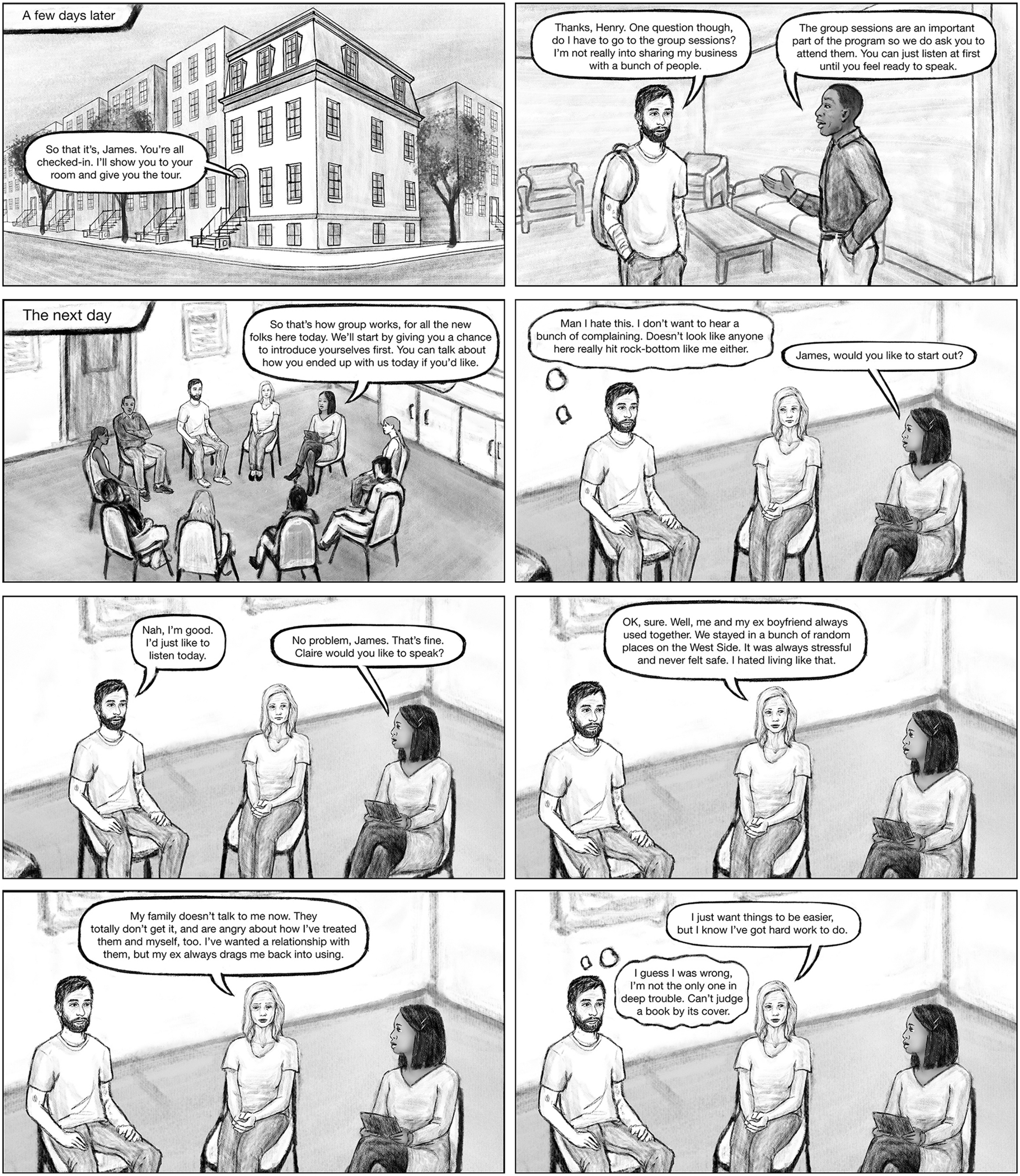

Images © 2022 JHU, illustrated by Lydia Gregg MA, CMI, FAMI

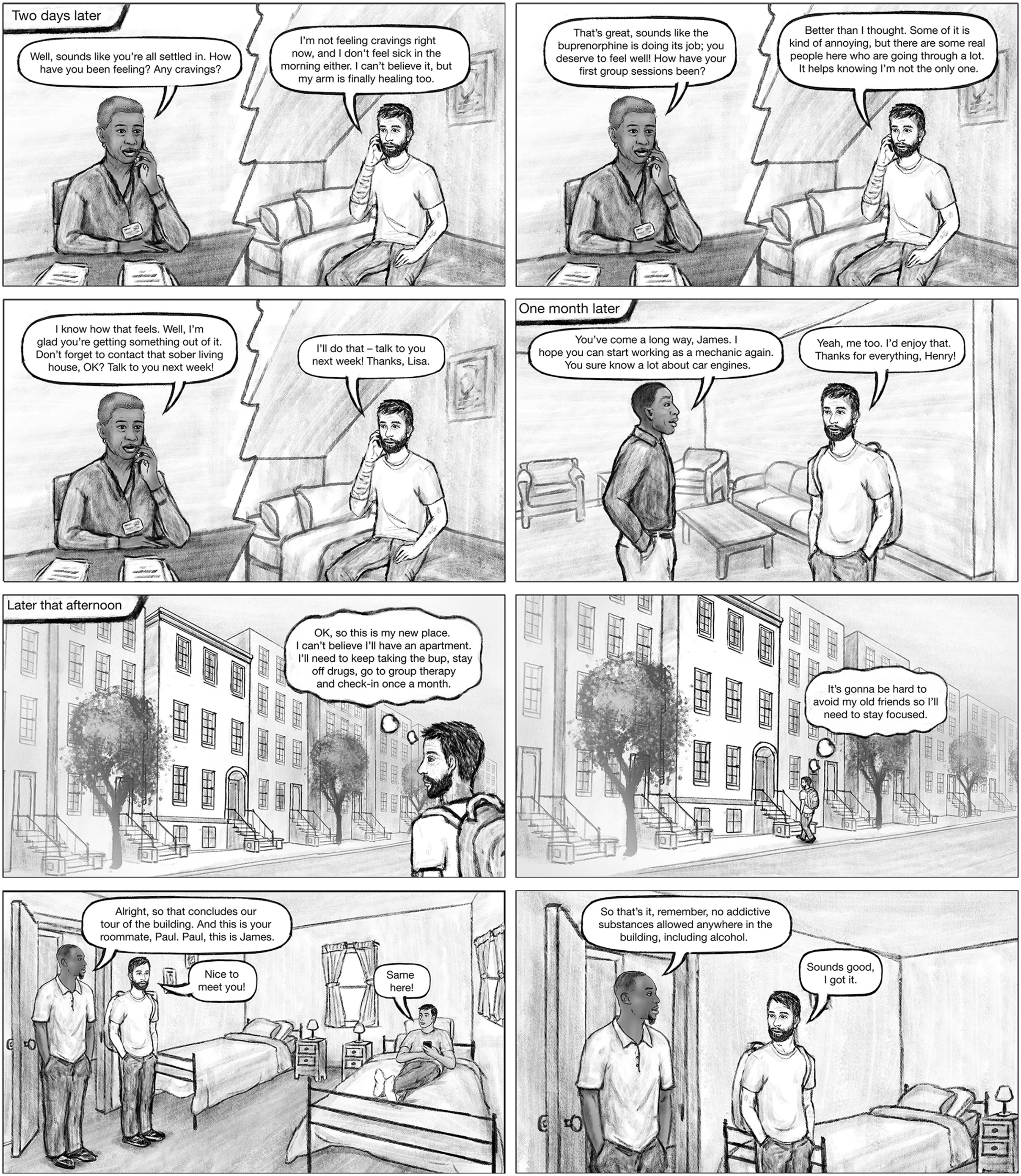

Images © 2022 JHU, illustrated by Lydia Gregg MA, CMI, FAMI

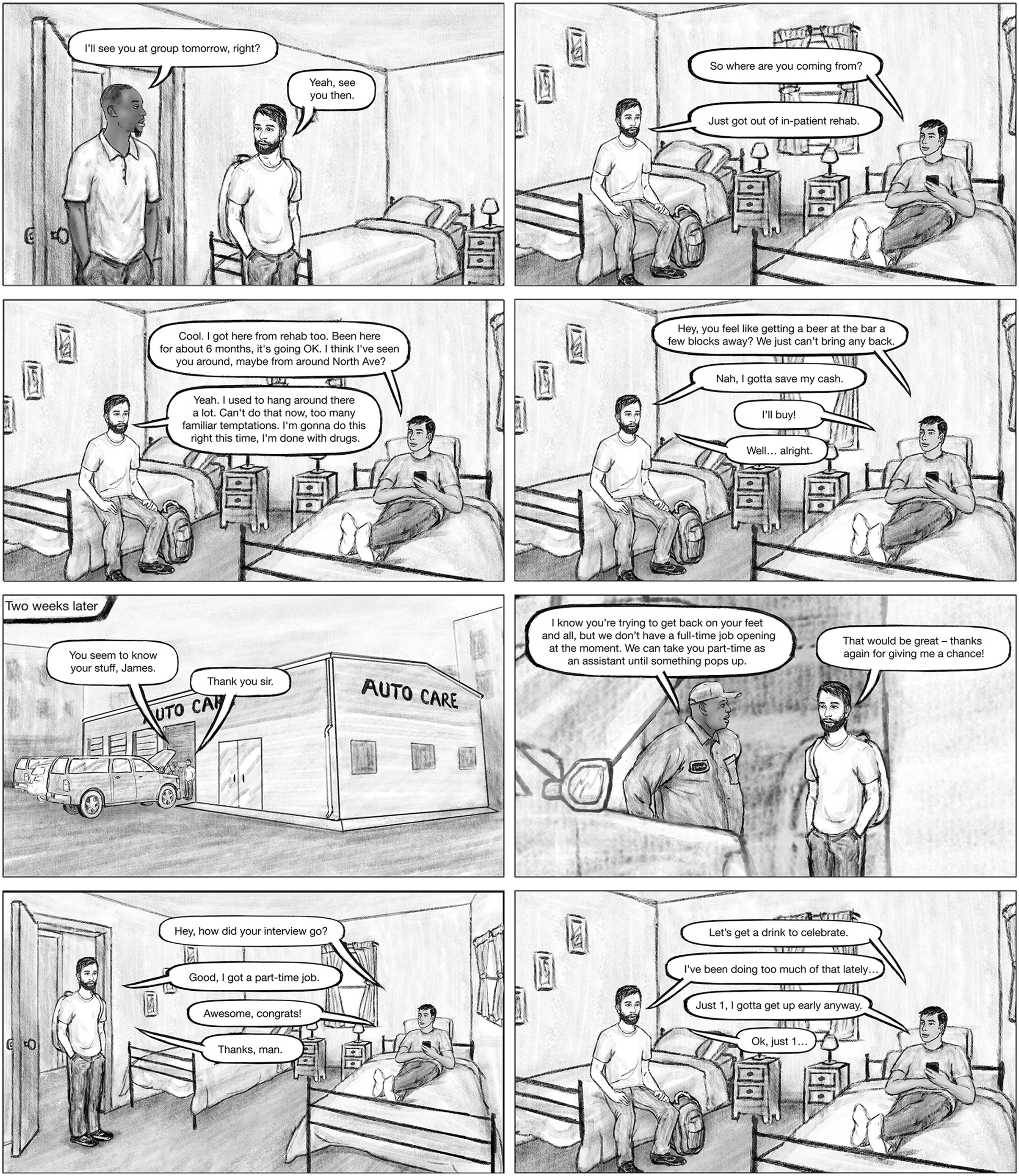

Images © 2022 JHU, illustrated by Lydia Gregg MA, CMI, FAMI

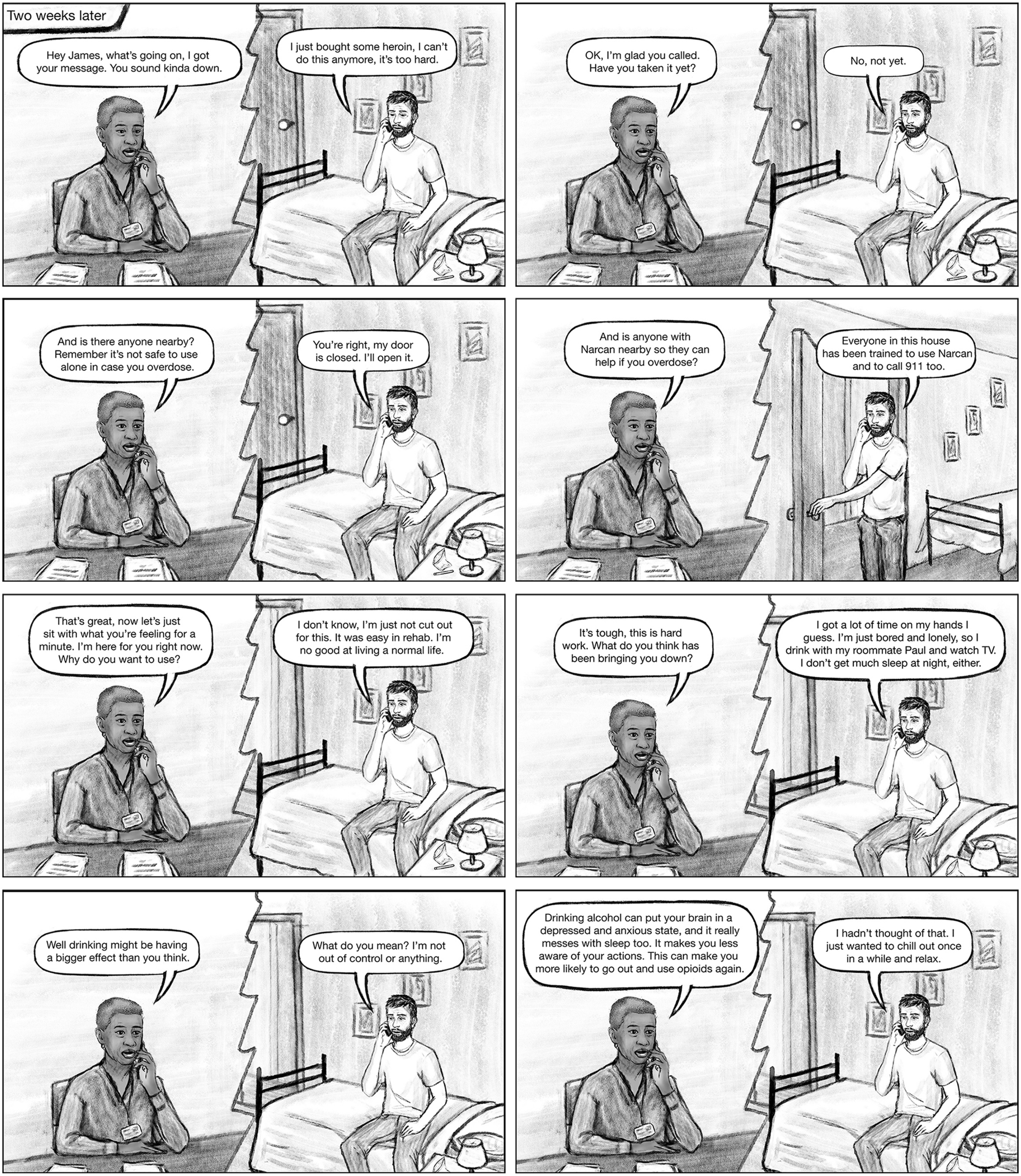

Images © 2022 JHU, illustrated by Lydia Gregg MA, CMI, FAMI

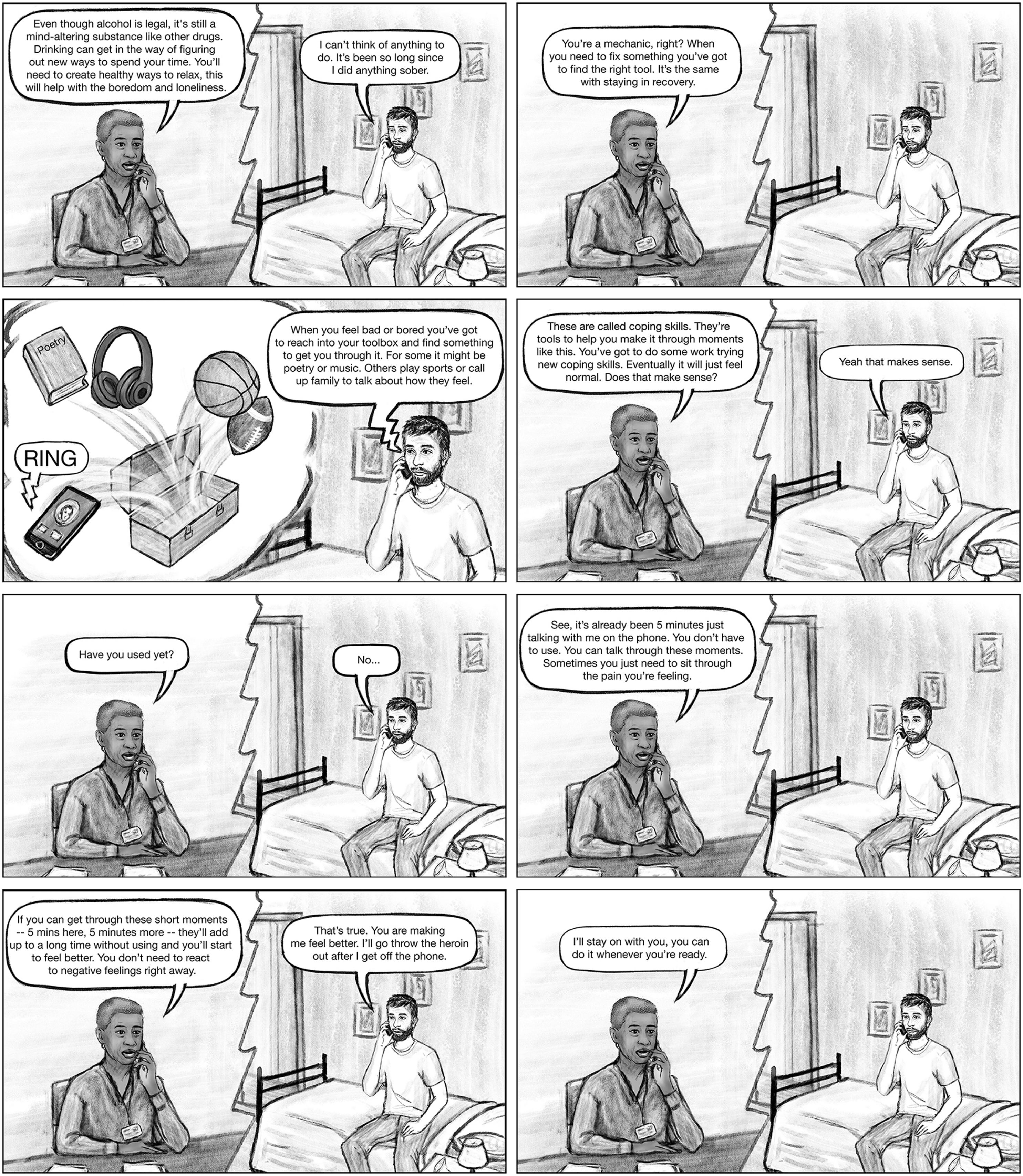

Images © 2022 JHU, illustrated by Lydia Gregg MA, CMI, FAMI

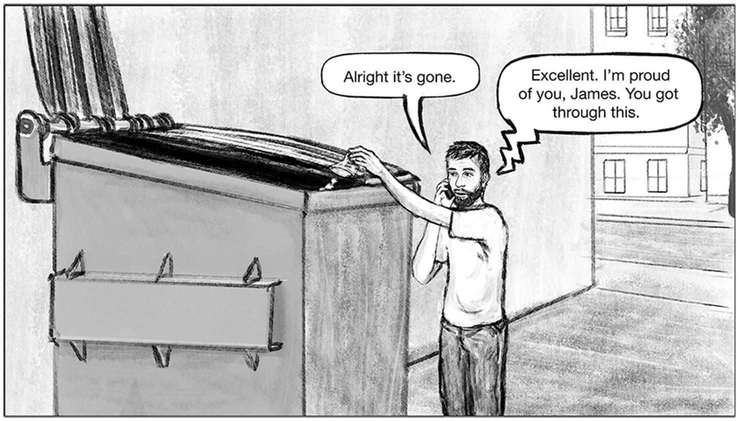

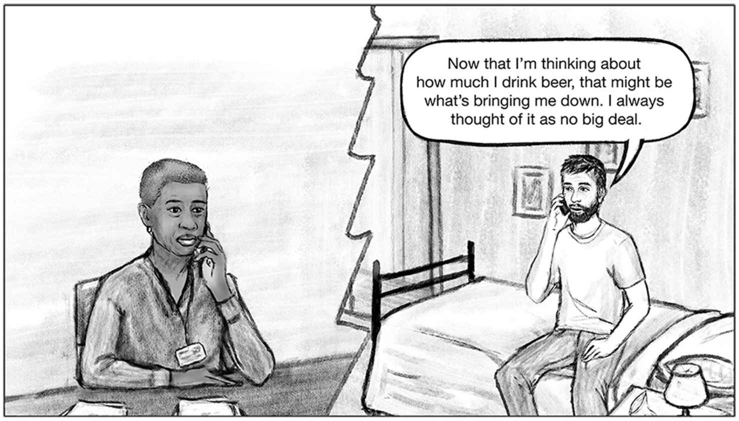

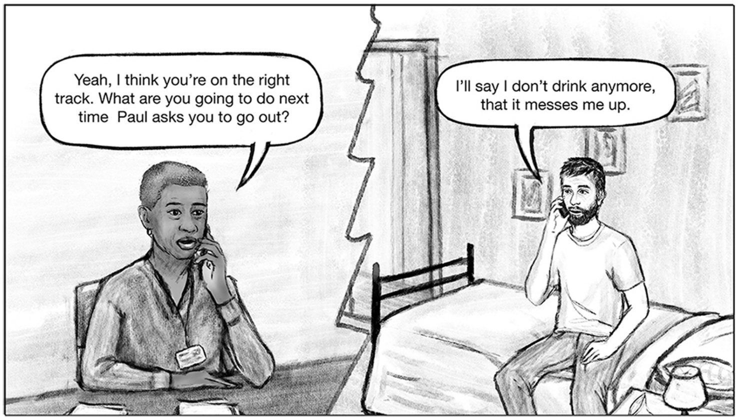

Images © 2022 JHU, illustrated by Lydia Gregg MA, CMI, FAMI
