# Supplementary material for: A Peer-Led, Narrative-Based, and Mobile-Supported Intervention in Opioid Use Disorder: Multiphase Qualitative and Longitudinal Observational Study
Source: JMIR Form Res. 2026 Feb 5;10:e82485. doi: 10.2196/82485 (PMC12875427; doi:10.2196/82485)
Supplement: Multimedia Appendix 7 [file formative-v10-e82485-s007.docx]

Supplement 7: Supplemental Tables

Table S1. Fisher’s tests for app use and peer navigator engagement and the association with opioid abstinence or use in the prior 30 days at follow-up, n=16

|  | Opioid abstinence, frequency (%), n=7 | Opioid use, frequency (%), n=9 | Odds ratio (95% CI) | p-value |
| --- | --- | --- | --- | --- |
| Used app^a^ | 7 (100.0%) | 4 (44.4%) | Inf (0.9, Inf) | 0.03 |
| Engaged with peer navigator^b^ | 7 (100.0%) | 3 (33.3%) | Inf (1.4, Inf) | 0.01 |

Abbreviations: CI: Confidence interval; Inf: Infinite.

^a^Odds ratio of those who used the app vs. did not use app

^b^Odds ratio of those who engaged the peer navigator vs. did not engage the peer navigator

Table S2. Fisher’s tests for app use and peer navigator engagement and the association with MOUD engagement or non-engagement in the prior 3 months at follow-up, n=16

|  | MOUD engagement, frequency (%), n=13 | No MOUD engagement, frequency (%), n=3 | Odds ratio (95% CI) | p-value |
| --- | --- | --- | --- | --- |
| Used app^a^ | 10 (76.9%) | 1 (33.3%) | 5.8 (0.2, 429.9) | 0.21 |
| Engaged with peer navigator^b^ | 9 (69.2%) | 1 (33.3%) | 4.1 (0.2, 293.6) | 0.52 |

Abbreviations: MOUD: Medications for opioid use disorder; CI: Confidence interval.

^a^Odds ratio of those who used the app vs. did not use app

^b^Odds ratio of those who engaged the peer navigator vs. did not engage the peer navigator
